# Age at Menarche and Cardiometabolic Health: A Sibling Analysis in the Scottish Family Health Study

**DOI:** 10.1161/JAHA.117.007780

**Published:** 2018-02-10

**Authors:** Maria C. Magnus, Debbie A. Lawlor, Stamatina Iliodromiti, Sandosh Padmanabhan, Scott M. Nelson, Abigail Fraser

**Affiliations:** ^1^ MRC Integrative Epidemiology Unit at the University of Bristol United Kingdom; ^2^ Department of Population Health Sciences Bristol Medical School University of Bristol United Kingdom; ^3^ NIHR Bristol Biomedical Research Centre at the University Hospitals Bristol NHS Foundation Trust University of Bristol United Kingdom; ^4^ Centre for Fertility and Health Norwegian Institute of Public Health Oslo Norway; ^5^ School of Medicine University of Glasgow United Kingdom; ^6^ Institute of Cardiovascular and Medical Sciences University of Glasgow United Kingdom

**Keywords:** cardiometabolic health, cardiovascular disease risk factors, menarche, sibships, Women, Risk Factors, Cardiovascular Disease, Epidemiology

## Abstract

**Background:**

Previous studies of age at menarche and cardiometabolic health report conflicting findings, and only a few could account for childhood characteristics. We aimed to estimate the associations of age at menarche with cardiovascular risk factors in unrelated women and within sister groups, under the assumption that within‐sibship estimates will be better adjusted for shared genetics and early life environment.

**Methods and Results:**

Our study included 7770 women, from 5984 sibships, participating in the GS:SFHS (Generation Scotland: Scottish Family Health Study). We used fixed‐ and between‐effects linear regression to estimate the associations within sister groups and between unrelated individuals, respectively. Within sibships, the mean difference between sisters with early menarche (≤11 years) and sisters with menarche at 12 to 13 years was 1.73 mm Hg (95% confidence interval [CI], −0.41 to 3.86) for systolic blood pressure, 1.26 mm Hg (95% CI, −0.02 to 2.55) for diastolic blood pressure, −0.06 nmol/L (95% CI, −0.11 to −0.02) for high‐density lipoprotein, 0.20 nmol/L (95% CI, 0.08–0.32) for non–high‐density lipoprotein, −0.34% (95% CI, −1.98 to 1.30) for glucose, 1.60 kg/m^2^ (95% CI, 0.92–2.28) for body mass index, and 2.75 cm (95% CI, 1.06–4.44) for waist circumference. There was weak evidence of associations between later menarche (14–15 or ≥16 years) and lower body mass index, waist circumference, and blood pressure. We found no strong evidence that estimates from within‐ and between‐sibship analyses differed (all *P* values >0.1). The associations with other cardiovascular risk factors were attenuated after adjustment for adult body mass index.

**Conclusions:**

Our results suggest that confounding by shared familial characteristics is unlikely to be a major driver of the association between early menarche and adverse cardiometabolic health but do not exclude confounding by individual‐level characteristics.

**Clinical Perspective**

**What Is New?**

- Associations of early menarche with cardiovascular risk factors were explained by body mass index in adulthood.
- Adverse cardiometabolic health in women with early menarche is not likely to be explained by shared familial characteristics such as genetics or childhood environment.

**What Are the Clinical Implications?**

- Earlier age at menarche is characteristic of women with more adverse cardiometabolic health.
- Having a healthy body mass index in adulthood could help diminish differences in cardiometabolic health related to age at menarche.

Early menarche is associated with reduced insulin sensitivity and higher glucose,^1^, ^2^, ^3^, ^4^, ^5^, ^6^, ^7^, ^8^, ^9^ higher triglycerides and cholesterol levels,^2^, ^6^, ^10^ higher blood pressure,^4^, ^5^, ^11^, ^12^ and greater waist circumference and body mass index (BMI).^4^, ^5^, ^13^, ^14^, ^15^ In line with these findings, there is some but less consistent evidence of an association between age at menarche and cardiovascular disease (CVD) events.^12^, ^16^, ^17^, ^18^ Mendelian randomization studies suggest causal effects of greater childhood BMI on early timing of menarche and of earlier menarche on higher adult BMI and CVD risk,^19^, ^20^ although genetic pleiotropy may at least partially explain these findings.^12^ The few observational analyses that were able to adjust for childhood adiposity found that the associations of age at menarche with adult cardiometabolic health were virtually completely attenuated, suggesting that childhood adiposity is a key confounder.^4^, ^14^ However, studies from populations in which childhood obesity is less prevalent, such as Korea,^5^, ^7^, ^8^ Bangladesh,^6^ China,^9^ and Brazil,^10^ also indicate an association between early menarche and worse cardiometabolic health.

Sibling studies controls for confounding (measured and unmeasured) by characteristics shared within families.^21^, ^22^ The underlying assumption of this approach is that siblings share identical or very similar early life environments in addition to, on average, half of their genetic architecture—often referred to as *fixed family characteristics*—and thus individual‐level confounding by characteristics that vary between siblings will be minimal.^23^ This approach has been used to explore the associations of intrauterine exposures, such as higher maternal BMI and gestational diabetes, with later offspring adiposity^24^ and of maternal age with perinatal outcomes,^25^ for which the key concern is family‐level socioeconomic confounding. Where the assumptions of the within‐sibship analysis are likely to hold, differences between associations seen in unrelated individuals and within sibling groups are interpreted as being due to residual individual‐person confounding in the former, whereas similarities in findings suggest that associations are not driven by shared genetic or early life environment.

In this study, we estimated and compared the associations of age at menarche with measures of cardiometabolic health in unrelated women and within sister groups. Our assumption was that adiposity and lifestyle characteristics before menarche (ie, up to age ≈10 years) were likely to be very similar among sisters and that there would be little individual‐level confounding by characteristics not shared among sisters up to this age.

## Methods

### Generation Scotland: Scottish Family Health Study

**Figure 1:**
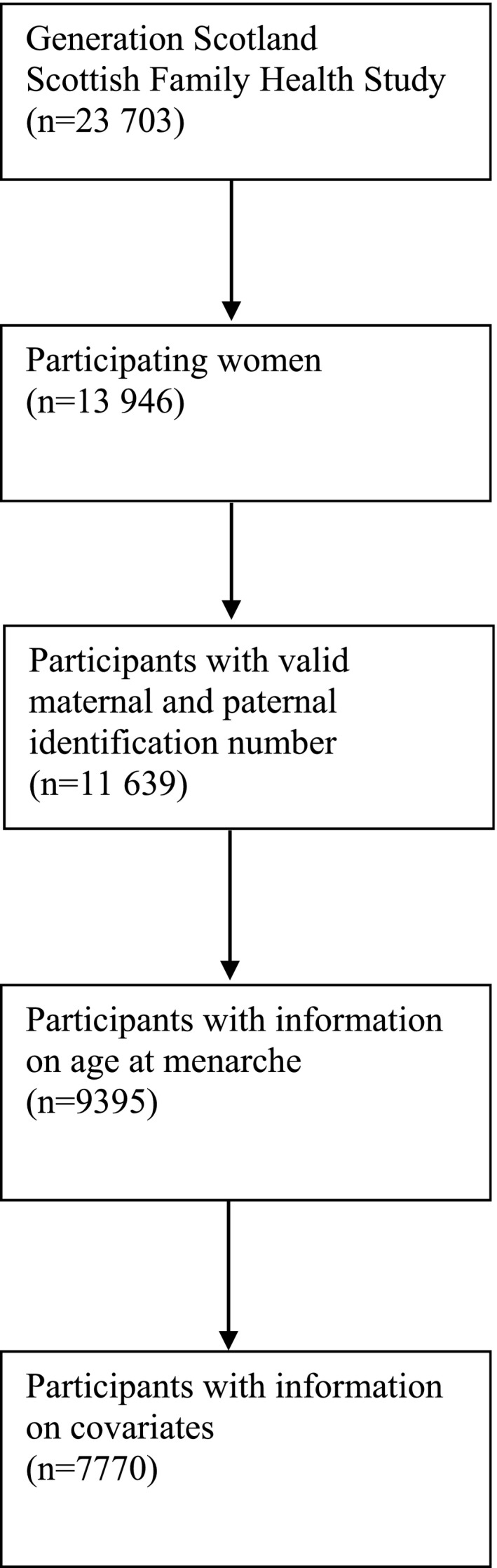
Illustration of the study sample, GS:SFHS (Generation Scotland: Scottish Family Health Study), 2006–2011.

This study included participants in the GS:SFHS (Generation Scotland: Scottish Family Health Study).^26^, ^27^ The data, analytical methods, and study materials will not be made available to other researchers for the purposes of reproducing the results or replicating the procedure. Individuals aged 35 to 65 years who were registered with collaborating general practitioners in Glasgow and Tayside (expanded to include Ayrshire, Arran, and northeast Scotland in 2010) were recruited between 2006 and 2011. All volunteers provided written informed consent and had to identify 1 first‐degree relative aged ≥18 years who would also consent to participate. Ethics approval was obtained by the National Health Service Tayside committee on research ethics (reference 05/s1401/89). Data collected included self‐reported information through questionnaires as well as clinical examinations and blood samples. The response rate was 5%, with 23 703 participants completing a preclinical questionnaire. Of the 13 946 women who completed the preclinical questionnaire, 11 639 had information on parental identification numbers needed to identify siblings, and 7770 had information on age at menarche and other covariates necessary for the current analysis (Figure 1). The study sample thus included 5984 sister groups. The number of women in each sibling group ranged from 1 (no participating sisters for comparison) to 6 (5 participating sisters for comparison). A total of 3327 women had at least 1 participating sister.

### Age at Menarche

The questionnaire used to obtain information about female reproductive health had 2 different versions. One version asked the woman to give her age in whole years when she had her first menstrual period, and the other version asked if her age at her first period was <8, 8 to 9, 10 to 11, 12 to 13, 14 to 15, 16 to 17, 18 to 19, ≥20, or not known. The new questionnaire was introduced in 2009 between July (Tayside) and October (Glasgow). The only difference between the groups that received the different questionnaires was the participation date. To allow for a nonlinear relationship, we categorized age at menarche as ≤11 years (early menarche), 12 to 13, 14 to 15, and ≥16 years (late menarche). The reference group in all analyses comprised those with an age at menarche of 12 to 13 years. There is some variation across studies in the definition of early menarche (≤10, ≤11, or ≤12 years) and late menarche (≥14, ≥15, or ≥16 years), likely influenced by the size and information available in the specific study, but our categorization is in line with commonly used cutoff values.^4^, ^5^, ^6^, ^7^, ^8^, ^10^, ^11^, ^14^, ^16^

### Cardiometabolic Health Outcomes

Cardiometabolic health was assessed by study nurses at recruitment. Systolic and diastolic blood pressure (mm Hg), calculated as the average of 2 measurements; BMI (weight in kg/height in m^2^); waist circumference (cm); and 12‐lead ECG were recorded (incorporated into a novel CVD risk prediction score). Total cholesterol, high‐density lipoprotein (HDL) cholesterol, and glucose were measured in serum using standard clinical assays. Non‐HDL cholesterol was calculated by subtracting HDL cholesterol from total cholesterol. Overall, 85% of the blood samples procured from participants were fasting (a minimum of 4 hours since the last meal). Furthermore, self‐reported information was available regarding diabetes mellitus in addition to the use of antihypertensive, lipid‐lowering, and antidiabetic drugs.

We calculated the 10‐year risk of CVD using 2 different risk scores. One was the Framingham 10‐year risk score, which includes age, total cholesterol, HDL cholesterol, systolic blood pressure, smoking, and diabetes mellitus.^28^ The second was a new validated 10‐year risk score for CVD from the NHANES (National Health and Nutrition Examination Survey) cohort that uses age and a range of measurements from ECG readings, including positive deflection of the T axis, negative deflection of the T axis, heart rate, and corrected QT interval.^29^ The risk scores were calculated only for individuals who were between 30 and 74 years of age (81% of those included in this analysis) because the original risk scores were generated for this age group. Individuals with self‐reported history of heart disease or stroke were excluded from the analysis of 10‐year risk of CVD.

### Potential Confounders

Additional self‐reported information on characteristics that could plausibly influence the associations of age at menarche with cardiometabolic health—and confound it—included age at recruitment (continuous), ethnicity (white versus other), qualifications (from none to college/university degree, including 7 categories in total), annual household income in pounds sterling (<10 000, 10 000–30 000, 30 000–50 000, 50 000–70 000, ≥70 000, prefer not to answer), number of pack‐years of smoking (none, 1–10, 11–20, >20), number of alcohol units consumed during the past week (none, 1–5, 6–10, >10 units), and number of hours of moderate or vigorous physical activity during the past week (≤1 hour, 1.1–3.0, 3.1–5.0, 5.1–10.0, 10.1–15.0, ≥15.1). Participants’ reports of parental history of CVD (heart disease, stroke, and/or high blood pressure) and diabetes mellitus were also considered.

### Statistical Analyses

We used fixed‐ and between‐effects linear regression to evaluate the associations of age at menarche with cardiometabolic health. Fixed‐effect linear regression provided the within‐sibships association, which is the association between age at menarche and cardiometabolic outcomes controlling for characteristics that are identical or very similar among sisters, including genetics, parental socioeconomic position, and childhood lifestyle and adiposity.^30^ The between‐sibships estimate was the association of age at menarche with cardiometabolic health in unrelated women. The estimate used data from all individuals but related the mean of the cardiometabolic measures within a cluster (group of sisters) to the mean age at menarche within a cluster (group of sisters).^30^ If the within‐ and between‐sibships estimates both provide evidence of an association, this suggests that the association between age at menarche and cardiometabolic health is not explained by unmeasured confounding due to genetic or environmental characteristics shared by siblings. To test whether the between‐ and within‐sibship estimates were different, we used a bootstrapping test with 5000 iterations. We also tested for departure from linearity in the association between age at menarche and cardiometabolic health, using a likelihood ratio test comparing a model with age at menarche as a categorical covariate and a model using age at menarche as a continuum.

We incrementally adjusted for age (model 1), ethnicity, educational qualifications, parental history of CVD, and parental history of diabetes mellitus (model 2). The multivariable analysis further adjusted blood pressure for use of antihypertensive drugs, cholesterol levels for lipid‐lowering drugs, and glucose for use of antidiabetic drugs. Potential confounders are common causes of the exposure and outcome. We did not have any direct measures of childhood socioeconomic position in GS:SFHS and thus had to rely on adult educational attainment as a proxy for childhood socioeconomic position. Under the assumption that there are genes that are common determinants of age at menarche and adverse cardiometabolic health, which is clearly the case for obesity‐related genes,^20^, ^31^ parental histories of CVD and diabetes mellitus were also conceptualized as confounders. We then explored further adjustment for adult lifestyle characteristics, including pack‐years of smoking, units of alcohol consumed during the past week, and number of hours of moderate or vigorous physical activity during the past week (model 3). These characteristics can be conceptualized as both potential confounders (due to tracking from childhood to adult life) and potential mediators, given evidence of associations between age at menarche and health‐related behaviors.^32^

We also conducted secondary analyses adjusting the other cardiometabolic health outcomes for adult BMI to further explore potential direct associations. Different sensitivity analyses included adjusting for adult household income (not adjusted for in the primary analysis because it also reflects the partner's contribution), excluding those on medications that could influence the outcomes of interest (for blood pressure, cholesterol, and glucose) and excluding women who had an age difference of >4 years with their only sibling for comparison (ie, restricting the within‐sibship analysis to sisters with an age difference of ≤4 years). This sensitivity analysis was done under the assumption that sisters who are closer in age are more likely to have a similar childhood environment. To examine the impact of nonfasting blood sampling on the associations, we reexamined the associations of age at menarche with HDL cholesterol, non‐HDL cholesterol, and glucose, excluding women with a nonfasting blood sample (n=1132) and unknown fasting status (n=321). We also conducted a sensitivity analysis excluding women of non‐European ethnicity (n=156).

The results presented are from a complete case analysis because it was not possible to conduct multiple imputation accounting for clustering, given the large number and small size of the sibling groups. All analyses were done using Stata version 14 (StataCorp).

## Results

Women included in the analyses were younger, were more likely to be white, had higher educational qualifications, had lower annual household income, and were more likely to have a family history of CVD than those excluded because of missing covariate information (Table S1). There was no difference in parental history of diabetes mellitus (Table S1). Of the women included in the analysis, 18% reported menarche at ≤11 years, whereas 52% were 12 to 13 years at menarche, 26% were 14 to 15 years at menarche, and 4% were ≥16 years at menarche. Age at menarche was associated with age at recruitment, qualifications, household income, parental history of diabetes mellitus, current use of antihypertensive medications, and, more weakly, with pack‐years of smoking, alcohol intake, and parental history of CVD (Table). A greater proportion of the variation in age at menarche, qualifications, and adult BMI, in addition to other adult lifestyle characteristics, was explained by variation within as opposed to between sibships (Table S2). Looking more closely at the level of concordance of these traits within sibships, there was a moderate to strong concordance for most traits (Table S3).

**Table 1:** Distribution of Background Characteristics by Age At Menarche, GS:SFHS, 2006–2011

Characteristics	Age At Menarche, y				
	≤11 (n=1395)	12–13 (n=4042)	14–15 (n=1993)	≥16 (n=340)	*P* Value
Age at baseline evaluation, y, mean±SD	44.4±13.6	43.6±13.7	45.2±13.8	47.0±12.9	<0.001
Ethnicity, n (%)					0.566
White	1365 (97.9)	3967 (98.1)	1947 (97.7)	335 (98.5)	
Other	30 (2.2)	75 (1.9)	46 (2.3)	5 (1.5)	
Qualifications, n (%)					<0.001
College/university degree	422 (30.3)	1451 (35.9)	680 (34.1)	98 (28.8)	
Other professional or technical qualification	300 (21.5)	789 (19.5)	394 (19.8)	76 (22.4)	
NVQ/HND/HNC or equivalent	131 (9.4)	345 (8.5)	154 (7.7)	31 (9.1)	
Higher grade	177 (12.7)	516 (12.8)	244 (12.2)	24 (7.1)	
Standard grade/O level/GCSE	184 (13.2)	486 (12.0)	248 (12.4)	49 (14.4)	
CSEs, school leavers certificate, other or no qualifications	181 (13.0)	455 (11.3)	273 (13.7)	62 (18.2)	
Annual household income, £, n (%)					0.005
<10 000	114 (8.2)	259 (6.4)	112 (5.6)	32 (9.4)	
10 000–30 000	414 (29.7)	1081 (26.7)	546 (27.4)	100 (29.4)	
30 000–50 000	335 (24.0)	1021 (25.3)	507 (25.4)	77 (22.7)	
50 000–70 000	172 (12.3)	579 (14.3)	306 (15.4)	37 (10.9)	
≥70 000	132 (9.5)	453 (11.2)	183 (9.2)	32 (9.4)	
Prefer not to answer	72 (5.2)	201 (5.0)	109 (5.5)	26 (7.7)	
Missing	156 (11.1)	448 (11.1)	230 (11.5)	36 (10.6)	
Pack‐years of smoking, n (%)					0.084
None	836 (59.9)	2566 (63.5)	1223 (61.4)	208 (61.2)	
1–10	247 (17.7)	703 (17.4)	366 (18.4)	57 (16.8)	
11–20	106 (7.6)	286 (7.1)	128 (6.4)	20 (5.9)	
≥20	206 (14.8)	487 (12.1)	276 (13.9)	55 (16.2)	
Number of alcohol units consumed during the past week, n (%)					0.083
None	330 (23.7)	813 (20.1)	425 (21.3)	64 (18.8)	
1–5	349 (25.0)	1001 (24.8)	457 (22.9)	93 (27.4)	
6–10	326 (23.4)	1039 (25.7)	486 (24.4)	79 (23.2)	
≥10	288 (20.7)	918 (22.7)	483 (24.2)	77 (22.7)	
Missing	102 (7.3)	271 (6.7)	142 (7.1)	27 (7.9)	
Number of hours of moderate or vigorous physical activity during the past week, n (%)					0.509
≤1	162 (11.6)	534 (13.2)	232 (11.6)	38 (11.2)	
1.1–3.0	296 (21.2)	875 (21.7)	415 (20.8)	65 (19.1)	
3.1–5.0	151 (10.8)	463 (11.5)	223 (11.2)	42 (12.4)	
5.1–10.0	300 (21.5)	734 (18.2)	383 (19.2)	69 (20.3)	
10.1–15.0	148 (10.6)	422 (10.4)	201 (10.1)	41 (12.1)	
≥15.1	225 (16.1)	689 (17.1)	360 (18.1)	55 (16.2)	
Missing	113 (8.1)	325 (8.0)	179 (9.0)	30 (8.8)	
Parental history of CVD, n (%)					0.088
No	461 (33.1)	1476 (36.5)	700 (35.1)	112 (32.9)	
Yes	934 (67.0)	2566 (63.5)	1293 (64.9)	228 (67.1)	
Parental history of diabetes mellitus, n (%)					0.037
No	1146 (82.2)	3423 (84.7)	1708 (85.7)	291 (85.6)	
Yes	249 (17.9)	619 (15.3)	285 (14.3)	49 (14.4)	
Use of antihypertensive medications, n (%)					0.002
No	1251 (89.7)	3733 (92.4)	1857 (93.2)	314 (92.4)	
Yes	144 (10.3)	309 (7.6)	136 (6.8)	26 (7.7)	
Use of lipid‐lowering medications, n (%)					0.376
No	1318 (94.5)	3863 (95.6)	1894 (95.0)	322 (94.7)	
Yes	77 (5.5)	179 (4.4)	99 (5.0)	18 (5.3)	
Use of antidiabetic medications, n (%)					0.745
No	1380 (98.9)	4004 (99.1)	1978 (99.3)	336 (98.8)	
Yes	15 (1.1)	38 (0.9)	15 (0.8)	4 (1.2)	

CSE indicates certificate of secondary education; CVD, cardiovascular disease; GCSE, general certificate of secondary educations; GS:SFHS, Generation Scotland: Scottish Family Health Study; HNC, higher national certificate; HND, higher national diploma; and NVQ,national vocational qualifications.

### Associations of Age at Menarche With Cardiometabolic Health Outcomes

**Figure 2:**
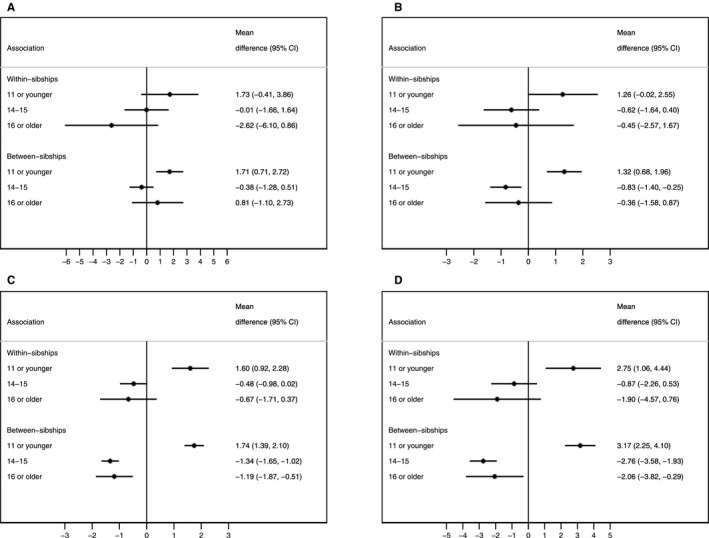
Adjusted associations of age at menarche with blood pressure and adiposity, GS:SFHS (Generation Scotland: Scottish Family Health Study), 2006–2011. The comparison group comprises women with an age at menarche of 12 or 13 years. A, Systolic blood pressure (mm Hg). B, Diastolic blood pressure (mm Hg). C, Body mass index. D, Waist circumference (cm). Adjusted for age, ethnicity, qualifications, parental history of cardiovascular disease, and parental history of diabetes mellitus. Blood pressure was further adjusted for use of antihypertensive drugs. CI indicates confidence interval.

**Figure 3:**
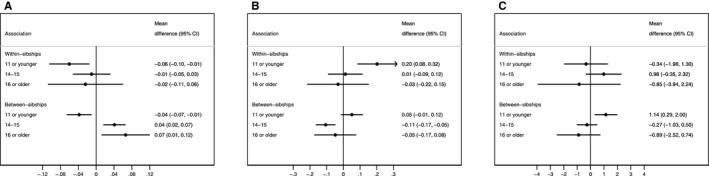
Adjusted associations of age at menarche with cholesterol and glucose, GS:SFHS (Generation Scotland: Scottish Family Health Study), 2006–2011. The comparison group comprises women with an age at menarche of 12 or 13 years. A, HDL cholesterol (mmol/L). B, Non‐HDL cholesterol (mmol/L). C, Glucose (mmol/L). Adjusted for age, ethnicity, qualifications, parental history of cardiovascular disease, and parental history of diabetes mellitus. Cholesterol levels further adjusted for lipid‐lowering drugs and glucose adjusted for use of antidiabetic drugs. CI indicates confidence interval; HDL, high‐density lipoprotein.

There was strong evidence of nonlinear association for most outcomes in both the between‐ and within‐sibship analyses (*P* values <0.01), with a few exceptions. Women with early menarche (≥11 years) had higher systolic and diastolic blood pressure and BMI and greater waist circumference compared with women with menarche at 12 to 13 years when examined both within and between sibships (Figure 2). Early menarche was also associated with lower HDL cholesterol and increased non‐HDL cholesterol both between and within sibships (Figure 3). There was no strong evidence for differences between the estimates from the between‐ and within‐sibship analyses from the bootstrapping tests (Table S4). The only exceptions were the estimates of the associations of age at menarche between 14 and 15 years (versus 12–13) with BMI and waist circumference, for which the inverse association tended to be greater when evaluated within sibships (*P*=0.02 and *P*=0.07, respectively; Table S4). Multivariable adjustment caused only modest changes in these associations, including adjustment for adult lifestyle characteristics (Table S4).

### Associations of Age at Menarche With 10‐Year Risk of CVD

**Figure 4:**
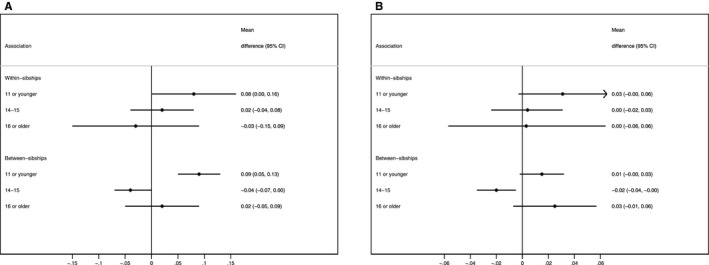
Adjusted association between age at menarche and 10‐year risk score of overall cardiovascular disease, GS:SFHS (Generation Scotland: Scottish Family Health Study), 2006–2011. The comparison group comprises women with an age at menarche of 12 or 13 years. A, Framingham risk score. B, NHANES (National Health and Nutrition Examination Survey) ECG risk score. The variables included in the Framingham risk score are age, total cholesterol, HDL cholesterol, systolic blood pressure, smoking, and diabetes mellitus. The information included in the NHANES ECG risk score included age, positive deflection of the T axis, negative deflection of the T axis, heart rate, and corrected QT interval. The associations are adjusted for age, ethnicity, qualifications, parental history of cardiovascular disease, and parental history of diabetes mellitus. CI indicates confidence interval.

The correlation between the Framingham and NHANES 10‐year CVD risk scores was 0.83. The likelihood ratio test comparing models including age at menarche as a categorical versus a continuous variable supported the presence of a nonlinear association between age at menarche and 10‐year risk of CVD (*P*<0.01). Early menarche was associated with higher 10‐year CVD risk using both scores compared with age at menarche of 12 to 13 years, which was consistent for both within‐ and between‐sibship estimates (Figure 4). Using the Framingham risk score, but not NHANES, age at menarche of ≥16 years was also associated with higher 10‐year CVD risk in models 1 and 2 but not in model 3 (which controlled for adult characteristics; Table S5).

### Sensitivity Analyses

Additional multivariable adjustment for adult household income did not change the associations (results available on request). Excluding those using antihypertensive medications from the analysis of blood pressure, those on lipid‐lowering medications from the analysis of cholesterol, and those on antidiabetic medications from the analysis of glucose yielded similar associations but wider confidence intervals (Table S6). When we adjusted the associations of age at menarche with other cardiometabolic outcomes for adult BMIs, all associations were attenuated and the confidence intervals included the null value (Tables S7 and S8). Restricting the within‐sibship analysis to sisters with an age difference of ≤4 years yielded associations of slightly greater magnitude (Tables S9 and S10). The sensitivity analysis excluding women of non‐European ethnicity yielded findings similar to the main analysis (Tables S11 and S12). Finally, excluding women with a nonfasting blood sample did not change the observed associations of age at menarche with HDL or non‐HDL cholesterol (Table S13). For glucose, the association with age at menarche of ≤11 years within sibships was of a slightly greater magnitude, whereas the association with menarche after 14 years within sibships was attenuated (Table S13). However, these changes in the associations did not change the overall conclusion.

## Discussion

In this sibship study, women who experienced early menarche (≤11 years) had a more adverse cardiometabolic profile and an increased 10‐year CVD risk score compared with women who experienced menarche at 12 to 13 years. The results were similar in unrelated women and within sister groups. Later menarche (14–15 and ≥16 years) was associated with lower BMI and waist circumference (both within and between sister groups) but not with other cardiometabolic health outcomes or the 10‐year risk of CVD.

These results suggest that associations found in this study and elsewhere^1^, ^2^, ^3^, ^4^, ^5^, ^6^, ^7^, ^8^, ^10^, ^12^, ^13^, ^15^ between early menarche and CVD risk factors and events are not explained by genetic or other characteristics shared by sisters. This interpretation requires a strong assumption: that there is little individual‐level confounding. If siblings differ to a greater extent with regard to distributions of potential confounders than to the exposure of interest, the within‐sibships analysis may be more biased than a standard analysis.^21^, ^33^ Consequently, a key underlying assumption is that childhood adiposity (a key potential confounder in this study), and other lifestyle characteristics, are more similar within sisters than between unrelated individuals, and that the concordance for these potential confounders is greater than the concordance for age at menarche. The GS:SFHS does not have any information on childhood environmental characteristics; therefore, we cannot directly test this assumption. We did find moderate to strong concordance within sibling groups for adult socioeconomic position and lifestyle characteristics, which indicates that the main confounders for this analysis are likely strongly correlated within siblings, since childhood lifestyle is assumed to be even more concordant within siblings than adult lifestyle. When we repeated the within‐sibships analyses among sisters with an age difference of up to 4 years, results were similar to the main analysis. Even though we found insufficient evidence to state that the estimates from the within‐ and between‐sibship analyses differed, this might be influenced by the sample size, and we cannot exclude the possibility that a larger sample could provide more conclusive evidence for, or against, an unconfounded causal effect of age at menarche with adverse cardiometabolic risk.

Siblings are widely assumed to experience a similar environment during early childhood, but we might speculate that they start to increasingly diverge around school age. However, evidence shows that physical activity has a strong heritable component during adolescence^34^, ^35^ and that family‐level characteristics play a more important role in determining children's sedentary time compared with school‐level characteristics.^36^ There is also a strong correlation in childhood adiposity among siblings, and having an obese elder sibling is associated with a 5‐fold increase in obesity in the younger sibling; the similarity is even greater among siblings of the same sex.^37^, ^38^

Our results could be influenced by selection bias due to the low participation rate in the GS:SFHS; however, the mean age at menarche in the cohort (13.1 years) is fairly similar to the average reported for women born between 1950 and 1980 from the Breakthrough Generations Study (≈12.7 years).^39^ Notably, we had information on age at menarche only in years and not months in GS:SFHS, and this could have resulted in a slight overestimation of the mean. It is also important to keep in mind that the low participation rate also reflects the unique sampling strategy of the cohort because participants were required to identify a family member who was also willing to participate. We cannot exclude the possibility that participation could be influenced by background characteristics associated with both the exposure and the outcome, such as childhood socioeconomic position and/or lifestyle characteristics. For example, the proportion of women who had a university degree in our analysis sample was greater than the national average identified in the 2011 Scottish census (34% versus 25%).^40^ This might have resulted in underestimation of the associations of interest.

We relied on self‐report of age at menarche a long time after the event occurred (median: 32 years; range: 5.5–59 years). This should not have resulted in substantial misclassification because previous studies have shown good validity of retrospectively recalled age at menarche.^34^ However, any misclassification in the exposure tends to exaggerate effects in within‐sibling analyses.^33^ Consequently, if there were substantial misclassification of age at menarche, it would have caused overestimation of the association with cardiometabolic health within sister groups, and contributed to the weak evidence of a difference in the associations within sister groups and between unrelated individuals. This possibility cannot be excluded. Finally, our study had limited power to evaluate associations with late menarche, given the relatively modest size of this group in the cohort.

Whether childhood adiposity is the sole driver of the associations of age at menarche with cardiometabolic health and CVD events, related to its strong inverse relationship with age at menarche,^41^, ^42^ remains to be determined. A limited number of studies were able to adjust for childhood characteristics when studying the associations of age at menarche with cardiometabolic health.^2^, ^4^, ^14^ Two studies that had data on BMI before menarche indicated that adjustment for childhood BMI virtually completely attenuated the association between age at menarche and adult BMI.^4^, ^14^ In this study and elsewhere, the associations of age at menarche with cardiometabolic outcomes were attenuated after adjustment for adult BMI.^4^ However, because BMI tracks across the life course, it is difficult to truly distinguish confounding (childhood BMI) from mediation (adult BMI) of the associations of age at menarche with other cardiometabolic health outcomes.

Greater confidence in causal inference from observational studies stems from consistent evidence across different studies and the use of different analytical approaches to address confounding and selection bias.^23^ The sibling comparison used in the current study is one such study‐design, but it is important to note that if its assumptions are violated, it may result in greater bias than conventional multivariable adjustment. Another increasingly popular approach is Mendelian randomization, which addresses unmeasured and residual confounding by using genetic polymorphisms as instrumental variables for the exposure of interest, based on their random allocation at conception resulting in their independence of confounding factors.^43^ However, the potential to use Mendelian randomization to study age at menarche in relation to cardiometabolic health is hampered by the number of overlapping genes associated with both age at menarche and adiposity.^20^, ^31^ Longitudinal studies with measures of adiposity before and after puberty have the potential to contribute valuable insight into the role of childhood adiposity in the associations of age at menarche with cardiometabolic health, with studies that have been able to do this suggesting that childhood BMI before puberty confounds any associations with adult BMI^4^, ^14^ and thus, potentially, with cardiometabolic risk.

In conclusion, early menarche is associated with an overall adverse cardiometabolic profile and a higher 10‐year risk score for CVD. The associations were similar when evaluated within sisters and between unrelated individuals, suggesting that confounding by shared familiar characteristics is unlikely to be a major driver of the association; but, this does not exclude counfounding by individual‐level characteristics.

## Sources of Funding

The data collection in Generation Scotland (http://www.generationscotland.org) is funded by the Chief Scientist Office (CZD/16/6) and the Scottish Family Funding Council (HR03006). Drs Magnus, Lawlor, and Fraser work in at the MRC Integrative Epidemiology Unit, which receives infrastructure funding from the UK Medical Research Council (MRC) (MC_UU_12013/5). Dr Fraser and Dr Magnus are funded by a UK MRC Fellowship awarded to Dr Fraser (MR/M009351/1). Dr Iliodromiti is funded by UK MRC a fellowship (MR/N015177/1). This study was supported by the National Institute for Health Research Biomedical Research Center at the University Hospitals Bristol National Health Service Foundation Trust and the University of Bristol. The views expressed in this publication are those of the authors and not necessarily those of the National Health Service, the National Institute for Health Research, or the Department of Health. This work was also supported by the Research Council of Norway Centres of Excellence funding scheme (project number 262700). These funding sources had no role in the design, collection, analysis, and interpretation of data; the writing of the article; or the decision to submit the article for publication.

## Disclosures

Dr Lawlor has received funding for biomarker research unrelated to this article from Roche Diagnostics and Ferring Pharmaceuticals. The remaining authors have no disclosures to report.

## Supporting information


**Table S1.** Background Characteristics Among Individuals Included and Excluded From Analyses Because of Missing Data, GS:SFHS (Generation Scotland: Scottish Family Health Study), 2006–2011
**Table S2.** Proportion of the Variation in Traits Explained by Variation Between and Within Sibships/Groups of Sisters, GS:SFHS (Generation Scotland: Scottish Family Health Study), 2006–2011
**Table S3.** Pairwise Discordance in Traits Between Sibships, GS:SFHS (Generation Scotland: Scottish Family Health Study), 2006–2011
**Table S4.** Association Between Age at Menarche and Cardiometabolic Health Outcomes, GS:SFHS (Generation Scotland: Scottish Family Health Study), 2006–2011
**Table S5.** Association Between Age at Menarche and 10‐Year Risk for Overall Cardiovascular Disease, GS:SFHS (Generation Scotland: Scottish Family Health Study), 2006–2011
**Table S6.** Associations of Age at Menarche With Blood Pressure, Cholesterol, and Glucose, Excluding Individuals on Medications That Might Influence the Outcomes, GS:SFHS (Generation Scotland: Scottish Family Health Study), 2006–2011
**Table S7.** Associations of Age at Menarche With Blood Pressure, Cholesterol, and Glucose After Adjustment for Body Mass Index, GS:SFHS (Generation Scotland: Scottish Family Health Study), 2006–2011
**Table S8.** Association Between Age at Menarche and 10‐Year Risk for Cardiovascular Disease After Adjustment for Adult Body Mass Index, GS:SFHS (Generation Scotland: Scottish Family Health Study), 2006–2011
**Table S9.** Association Between Age at Menarche and Cardiometabolic Health After Restricting the Analysis to Sibships With Up to 4 Years’ Age Difference Between Sisters, GS:SFHS (Generation Scotland: Scottish Family Health Study), 2006–2011
**Table S10.** Association Between Age at Menarche and 10‐Year Risk of Cardiovascular Disease After Restricting the Analysis to Sibships With Up to 4 Years’ Age Difference Between Sisters, GS:SFHS (Generation Scotland: Scottish Family Health Study), 2006–2011
**Table S11.** Association Between Age at Menarche and Cardiometabolic Health Outcomes Excluding Individuals of Non‐European Ethnicity, GS:SFHS (Generation Scotland: Scottish Family Health Study), 2006–2011
**Table S12.** Association Between Age at Menarche and 10‐Year Risk for Overall Cardiovascular Disease Excluding Individuals of Non‐European Ethnicity, GS:SFHS (Generation Scotland: Scottish Family Health Study), 2006–2011
**Table S13.** Association Between Age at Menarche and Cardiometabolic Health Outcomes Excluding Individuals With Nonfasting Blood Samples, GS:SFHS (Generation Scotland: Scottish Family Health Study), 2006–2011
