# Supplementary material for: Age at Menarche and Cardiometabolic Health: A Sibling Analysis in the Scottish Family Health Study
Source: J Am Heart Assoc. 2018 Feb 10;7(4):e007780. doi: 10.1161/JAHA.117.007780 (PMC5850196; doi:10.1161/JAHA.117.007780)
Supplement: Supplementary file 1 — Table S1. Background Characteristics Among Individuals Included and Excluded From Analyses Because of Missing Data, GS:SFHS (Generation Scotland: Scottish Family Health Study), 2006–2011 Table S2. Proportion of the Variation in Traits Explained by Variation Between and Within Sibships/Groups of Sisters, GS:SFHS (Generation Scotland: Scottish Family Health Study), 2006–2011 Table S3. Pairwise Discordance in Traits Between Sibships, GS:SFHS (Generation Scotland: Scottish Family Health Study), 2006–2011 Table S4. Association Between Age at Menarche and Cardiometabolic Health Outcomes, GS:SFHS (Generation Scotland: Scottish Family Health Study), 2006–2011 Table S5. Association Between Age at Menarche and 10‐Year Risk for Overall Cardiovascular Disease, GS:SFHS (Generation Scotland: Scottish Family Health Study), 2006–2011 Table S6. Associations of Age at Menarche With Blood Pressure, Cholesterol, and Glucose, Excluding Individuals on Medications That Might Influence the Outcomes, GS:SFHS (Generation Scotland: Scottish Family Health Study), 2006–2011 Table S7. Associations of Age at Menarche With Blood Pressure, Cholesterol, and Glucose After Adjustment for Body Mass Index, GS:SFHS (Generation Scotland: Scottish Family Health Study), 2006–2011 Table S8. Association Between Age at Menarche and 10‐Year Risk for Cardiovascular Disease After Adjustment for Adult Body Mass Index, GS:SFHS (Generation Scotland: Scottish Family Health Study), 2006–2011 Table S9. Association Between Age at Menarche and Cardiometabolic Health After Restricting the Analysis to Sibships With Up to 4 Years’ Age Difference Between Sisters, GS:SFHS (Generation Scotland: Scottish Family Health Study), 2006–2011 Table S10. Association Between Age at Menarche and 10‐Year Risk of Cardiovascular Disease After Restricting the Analysis to Sibships With Up to 4 Years’ Age Difference Between Sisters, GS:SFHS (Generation Scotland: Scottish Family Health Study), 2006–2011 Table S11. Association Between Age at Men [file JAH3-7-e007780-s001.pdf]

# SUPPLEMENTAL MATERIAL

**Table S1.** Background Characteristics Among Individuals Included and Excluded from Analyses Due to Missing Data, the Scottish Family Health Study, 2006-2011.

| Characteristics | Excluded (n= 6,176) | | Included (n=7,770) | | P-value |
|---|---|---|---|---|---|
| | n/mean | %/SD | n/mean | %/SD | |
| Age at baseline evaluation | 52.1 | 16.1 | 44.3 | 13.7 | <0.001 |
| Ethnicity | | | | | <0.001 |
| White | 5,691 | 95.9 | 7,614 | 98.0 | |
| Other | 246 | 4.1 | 156 | 2.0 | |
| Missing | 239 | | 0 | | |
| Qualifications | | | | | <0.001 |
| College/University degree | 1,594 | 31.0 | 2,651 | 34.1 | |
| Other professional or technical qualification | 826 | 16.1 | 1,559 | 20.1 | |
| NVQ/HND/HNC or equivalent | 472 | 9.2 | 661 | 8.5 | |
| Higher Grade | 465 | 9.1 | 961 | 12.4 | |
| Standard Grade/O Level/GCSE | 590 | 11.5 | 967 | 12.5 | |
| CSEs, School leavers certificate, other or no qualifications | 1,193 | 23.2 | 971 | 12.5 | |
| Missing | 1,036 | | 0 | | |
| Annual household income, pounds | | | | | <0.001 |
| <10,000 | 509 | 10.4 | 517 | 7.5 | |
| 10,000-30,000 | 1,591 | 32.4 | 2,141 | 31.0 | |
| 30,000-50,000 | 1,151 | 23.5 | 1,940 | 28.1 | |
| 50,000-70,000 | 627 | 12.8 | 1,094 | 15.9 | |
| 70,000+ | 510 | 10.4 | 800 | 11.6 | |
| Prefer not to answer | 516 | 10.5 | 408 | 5.9 | |
| Missing | 1,272 | | 870 | | |
| Parental history of CVD | | | | | <0.001 |
| No | 1,260 | 32.0 | 2,749 | 35.4 | |
| Yes | 2,673 | 68.0 | 5,021 | 64.6 | |
| Missing | 2,243 | | 0 | | |
| Parental history of diabetes | | | | | 0.143 |
| No | 3,365 | 85.6 | 6,568 | 84.5 | |
| Yes | 568 | 14.4 | 1,202 | 15.5 | |
| Missing | 2,243 | | 0 | | |

CVD=cardiovascular disease.

Percentages and test of differences in distributions among those with and without the necessary follow-up information are based on observed values and missing categories are not included.

**Table S2.** Proportion of the Variation in Traits Explained by Variation Between and Within Sibships/Groups of Sisters, the Scottish Family Health Study, 2006-2011.

| Characteristics | Proportion explained by variation between sibships | Proportion explained by variation within sibships |
|---|---|---|
| Age (years) | | |
| Age at menarche (years) | 0.27 | 0.73 |
| Qualifications (ordered from CSE/school leavers to college/university degree) | 0.40 | 0.60 |
| Current smoking at recruitment (yes versus no) | 0.37 | 0.63 |
| Hours of moderate/vigorous physical activity in the past week | 0.48 | 0.52 |
| Units of alcohol consumed in the past week | 0.07 | 0.93 |
| Adult BMI | 0.31 | 0.69 |

BMI=body-mass index.

The proportion of variation explained by variation between sibships/groups of sisters estimated using the intra-class correlation coefficient from a random effects linear/logistic regression model.

**Table S3.** Pairwise Discordance in Traits Between Sibships, the Scottish Family Health Study, 2006-2011.

| Characteristics | Number of pairs | % |
|---|---|---|
| Difference in age (years)<br>Number of sibling groups=1,541 (Number of women=3,327) | | |
| 0-2 | 584 | 37.9 |
| 3-4 | 464 | 30.1 |
| 5-6 | 219 | 14.2 |
| More than 6 | 274 | 17.8 |
| Difference in age at menarche (years)<br>Number of sibling groups=1,541 (Number of women=3,327) | | |
| 0 | 582 | 37.8 |
| 1 | 331 | 21.5 |
| 2 | 440 | 28.6 |
| 3 | 93 | 6.0 |
| More than 3.0 | 95 | 6.2 |
| Discordance in college/university degree<br>Number of sibling groups=1,541(Number of women=3,327) | | |
| No | 1,139 | 73.9 |
| Yes | 402 | 26.1 |
| Discordance in current smoking at recruitment<br>Number of groups= 1,520 (Number of women= 3,280) | | |
| No | 1,235 | 81.3 |
| Yes | 285 | 18.7 |
| Difference in hours of moderate/vigorous physical activity in the past week<br>Number of sibling groups= 1,332 (Number of women= 2,863) | | |
| 0-2.9 | 524 | 39.3 |
| 3.0-5.9 | 310 | 23.2 |
| 6.0 or more | 498 | 37.4 |
| Difference in units of alcohol consumed in the past week<br>Number of sibling groups= 1,378 (Number of women= 2,961) | | |
| 0-1 | 304 | 22.1 |
| 2-3 | 297 | 21.6 |
| 4-5 | 204 | 14.8 |
| 6 or more | 573 | 41.6 |
| Difference in adult body-mass index<br>Number of sibling groups= 1,513 (Number of women= 3,265) | | |
| 0-2 | 582 | 38.5 |
| 3-4 | 318 | 21.0 |
| 5-6 | 233 | 15.4 |
| More than 6 | 380 | 25.1 |

**Table S4.** Association Between Age at Menarche and Cardiometabolic Health Outcomes, the Scottish Family Health Study, 2006-2011.

| Outcome | Model | Age at menarche | N | Mean/Median | SD/Range | Within-sibships association | | Between-sibships association | | Bootstrap p-value for the difference in the within and between sibship association |
|---|---|---|---|---|---|---|---|---|---|---|
| | | | | | | β | 95% CI | β | 95% CI | |
| Systolic blood pressure (mmHg) | Model 1 | 11 or younger | 1,392 | 127.5 | 17.2 | 1.97 | -0.16, 4.10 | 2.08 | 1.06, 3.10 | 0.99 |
| | | 12-13 | 4,034 | 124.9 | 16.5 | Ref | | Ref | | NA |
| | | 14-15 | 1,989 | 125.6 | 17.6 | -0.02 | -1.67, 1.62 | -0.47 | -1.37, 0.44 | 0.67 |
| | | 16 or higher | 339 | 126.6 | 18.0 | -2.50 | -5.98, 0.97 | 0.76 | -1.18, 2.69 | 0.33 |
| | Model 2 | 11 or younger | 1,392 | 127.5 | 17.2 | 1.73 | -0.41, 3.86 | 1.71 | 0.71, 2.72 | 0.94 |
| | | 12-13 | 4,034 | 124.9 | 16.5 | Ref | | Ref | | NA |
| | | 14-15 | 1,989 | 125.6 | 17.6 | -0.01 | -1.66, 1.64 | -0.38 | -1.28, 0.51 | 0.74 |
| | | 16 or higher | 339 | 126.6 | 18.0 | -2.62 | -6.10, 0.86 | 0.81 | -1.10, 2.73 | 0.31 |
| | Model 3 | 11 or younger | 1,189 | 127.3 | 17.2 | 2.26 | -0.26, 4.78 | 1.74 | 0.68, 2.80 | 0.78 |
| | | 12-13 | 3,474 | 124.7 | 16.3 | Ref | | Ref | | NA |
| | | 14-15 | 1,687 | 125.1 | 17.3 | 0.44 | -1.45, 2.32 | -0.41 | -1.35, 0.53 | 0.54 |
| | | 16 or higher | 285 | 126.1 | 17.4 | -2.74 | -6.52, 1.04 | 0.31 | -1.69, 2.32 | 0.34 |
| Diastolic blood pressure (mmHg) | Model 1 | 11 or younger | 1,392 | 79.2 | 10.1 | 1.39 | 0.11, 2.67 | 1.48 | 0.83, 2.12 | 0.98 |
| | | 12-13 | 4,034 | 77.5 | 9.9 | Ref | | Ref | | NA |
| | | 14-15 | 1,989 | 77.1 | 10.0 | -0.62 | -1.64, 0.41 | -0.86 | -1.44, -0.28 | 0.70 |
| | | 16 or higher | 339 | 77.8 | 10.1 | -0.38 | -2.49, 1.73 | -0.38 | -1.62, 0.85 | 0.86 |
| | Model 2 | 11 or younger | 1,392 | 79.2 | 10.1 | 1.26 | -0.02, 2.55 | 1.32 | 0.68, 1.96 | 0.99 |
| | | 12-13 | 4,034 | 77.5 | 9.9 | Ref | | Ref | | NA |
| | | 14-15 | 1,989 | 77.1 | 10.0 | -0.62 | -1.64, 0.40 | -0.83 | -1.40, -0.25 | 0.75 |
| | | 16 or higher | 339 | 77.8 | 10.1 | -0.45 | -2.57, 1.67 | -0.36 | -1.58, 0.87 | 0.89 |
| | Model 3 | 11 or younger | 1,189 | 79.2 | 10.1 | 1.74 | 0.22, 3.25 | 1.39 | 0.71, 2.08 | 0.75 |
| | | 12-13 | 3,474 | 77.4 | 9.8 | Ref | | Ref | | NA |
| | | 14-15 | 1,687 | 77.0 | 10.1 | -0.37 | -1.52, 0.78 | -0.66 | -1.27, -0.05 | 0.67 |
| | | 16 or higher | 285 | 77.8 | 10.3 | -0.93 | -3.40, 1.54 | -0.06 | -1.36, 1.24 | 0.74 |
| HDL cholesterol, (mmol/L) | Model 1 | 11 or younger | 1,338 | 1.537 | 0.404 | -0.064 | -0.109, -0.019 | -0.045 | -0.074, -0.017 | 0.65 |
| | | 12-13 | 3,851 | 1.591 | 0.418 | Ref | | Ref | | NA |
| | | 14-15 | 1,897 | 1.627 | 0.405 | -0.009 | -0.050, 0.033 | 0.040 | 0.014, 0.065 | 0.13 |
| | | 16 or higher | 326 | 1.646 | 0.432 | -0.025 | -0.109, 0.060 | 0.058 | 0.004, 0.113 | 0.21 |

| | | | | | | | | | | |
|---|---|---|---|---|---|---|---|---|---|---|
| | Model 2 | 11 or younger | 1,338 | 1.537 | 0.404 | -0.060 | -0.105, -0.015 | -0.038 | -0.066, -0.010 | 0.54 |
| | | 12-13 | 3,851 | 1.591 | 0.418 | Ref | | Ref | | NA |
| | | 14-15 | 1,897 | 1.627 | 0.405 | -0.010 | -0.052, 0.032 | 0.041 | 0.016, 0.066 | 0.11 |
| | | 16 or higher | 326 | 1.646 | 0.432 | -0.024 | -0.108, 0.060 | 0.066 | 0.012, 0.120 | 0.18 |
| | Model 3 | 11 or younger | 1,146 | 1.547 | 0.405 | -0.042 | -0.093, 0.008 | -0.038 | -0.067, -0.009 | 0.96 |
| | | 12-13 | 3,315 | 1.604 | 0.415 | Ref | | Ref | | NA |
| | | 14-15 | 1,615 | 1.639 | 0.408 | -0.009 | -0.057, 0.039 | 0.037 | 0.011, 0.063 | 0.22 |
| | | 16 or higher | 276 | 1.674 | 0.437 | -0.017 | -0.105, 0.071 | 0.061 | 0.005, 0.116 | 0.24 |
| Non-HDL cholesterol (mmol/L) | Model 1 | 11 or younger | 1,338 | 3.638 | 1.065 | 0.196 | 0.073, 0.319 | 0.063 | -0.004, 0.131 | 0.20 |
| | | 12-13 | 3,851 | 3.517 | 1.050 | Ref | | Ref | | NA |
| | | 14-15 | 1,897 | 3.498 | 1.031 | 0.021 | -0.087, 0.129 | -0.100 | -0.160, -0.040 | 0.13 |
| | | 16 or higher | 326 | 3.583 | 1.008 | -0.035 | -0.235, 0.166 | -0.030 | -0.158, 0.099 | 0.97 |
| | Model 2 | 11 or younger | 1,338 | 3.638 | 1.065 | 0.202 | 0.084, 0.321 | 0.051 | -0.015, 0.117 | 0.13 |
| | | 12-13 | 3,851 | 3.517 | 1.050 | Ref | | Ref | | NA |
| | | 14-15 | 1,897 | 3.498 | 1.031 | 0.012 | -0.092, 0.115 | -0.106 | -0.165, -0.047 | 0.14 |
| | | 16 or higher | 326 | 3.583 | 1.008 | -0.032 | -0.217, 0.153 | -0.048 | -0.174, 0.077 | 0.88 |
| | Model 3 | 11 or younger | 1,146 | 3.629 | 1.068 | 0.142 | 0.009, 0.274 | 0.068 | -0.002, 0.137 | 0.49 |
| | | 12-13 | 3,315 | 3.491 | 1.039 | Ref | | Ref | | NA |
| | | 14-15 | 1,615 | 3.462 | 1.021 | -0.013 | -0.132, 0.105 | -0.092 | -0.154, -0.030 | 0.37 |
| | | 16 or higher | 276 | 3.539 | 1.001 | 0.071 | -0.281, 0.139 | -0.070 | -0.201, 0.061 | 0.96 |
| Glucose, (mmol/L)* | Model 1 | 11 or younger | 1,317 | 4.6 | 4.3, 4.9 | -0.732 | -2.418, 0.954 | 1.349 | 0.456, 2.241 | 0.11 |
| | | 12-13 | 3,814 | 4.5 | 4.3, 4.8 | Ref | | Ref | | NA |
| | | 14-15 | 1,876 | 4.6 | 4.3, 4.8 | 0.879 | -0.504, 2.262 | -0.386 | -1.185, 0.413 | 0.29 |
| | | 16 or higher | 327 | 4.6 | 4.3, 4.8 | -0.761 | -3.803, 2.281 | -0.853 | -2.553, 0.846 | 0.91 |
| | Model 2 | 11 or younger | 1,317 | 4.6 | 4.3, 4.9 | -0.338 | -1.975, 1.298 | 1.143 | 0.287, 1.999 | 0.23 |
| | | 12-13 | 3,814 | 4.5 | 4.3, 4.8 | Ref | | Ref | | NA |
| | | 14-15 | 1,876 | 4.6 | 4.3, 4.8 | 0.983 | -0.352, 2.318 | -0.266 | -1.032, 0.499 | 0.27 |
| | | 16 or higher | 327 | 4.6 | 4.3, 4.8 | -0.849 | -3.941, 2.243 | -0.892 | -2.521, 0.737) | 0.89 |
| | Model 3 | 11 or younger | 1,126 | 4.6 | 4.3, 4.9 | -0.022 | -2.002, 1.958 | 1.006 | 0.125, 1.888 | 0.43 |
| | | 12-13 | 3,282 | 4.5 | 4.3, 4.8 | Ref | | Ref | | NA |
| | | 14-15 | 1,599 | 4.6 | 4.3, 4.8 | 1.534 | 0.021, 3.047 | -0.212 | -0.999, 0.576 | 0.17 |
| | | 16 or higher | 276 | 4.6 | 4.3, 4.8 | -2.585 | -6.208, 1.038 | -0.721 | -2.396, 0.953 | 0.54 |
| Body-mass index ($kg/m^2$) | Model 1 | 11 or younger | 1,374 | 28.081 | 6.170 | 1.687 | 0.997, 2.377 | 1.871 | 1.509, 2.232 | 0.64 |
| | | 12-13 | 3,997 | 26.107 | 5.195 | Ref | | Ref | | NA |
| | | 14-15 | 1,975 | 25.048 | 4.771 | -0.477 | -0.976, 0.022 | -1.343 | -1.664, -1.021 | 0.02 |
| | | 16 or higher | 334 | 25.424 | 5.398 | -0.650 | -1.693, 0.392 | -1.060 | -1.749, -0.371 | 0.73 |

| | | | | | | | | | | |
|---|---|---|---|---|---|---|---|---|---|---|
| | Model 2 | 11 or younger | 1,374 | 28.081 | 6.170 | 1.598 | 0.919, 2.278 | 1.742 | 1.386, 2.098 | 0.70 |
| | | 12-13 | 3,997 | 26.107 | 5.195 | Ref | | Ref | | NA |
| | | 14-15 | 1,975 | 25.048 | 4.771 | -0.478 | -0.981, 0.024 | -1.338 | -1.655, -1.022 | 0.03 |
| | | 16 or higher | 334 | 25.424 | 5.398 | -0.670 | -1.705, 0.366 | -1.187 | -1.865, -0.509 | 0.65 |
| | Model 3 | 11 or younger | 1,146 | 3.629 | 1.068 | 1.691 | 0.942, 2.440 | 1.663 | 1.295, 2.031 | 0.94 |
| | | 12-13 | 3,315 | 3.491 | 1.039 | Ref | | Ref | | NA |
| | | 14-15 | 1,615 | 3.462 | 1.021 | -0.620 | -1.172, -0.068 | -1.249 | -1.576, -0.921 | 0.16 |
| | | 16 or higher | 276 | 3.539 | 1.001 | -1.411 | -2.511, -0.311 | -0.959 | -1.658, -0.260 | 0.56 |
| Waist circumference, (cm) | Model 1 | 11 or younger | 1,364 | 88.20 | 15.30 | 2.907 | 1.200, 4.614 | 3.530 | 2.590, 4.472 | 0.55 |
| | | 12-13 | 3,981 | 84.48 | 13.80 | Ref | | Ref | | NA |
| | | 14-15 | 1,966 | 82.53 | 12.71 | -0.847 | -2.240, 0.546 | -2.723 | -3.560, -1.887 | 0.07 |
| | | 16 or higher | 335 | 83.66 | 14.49 | -1.903 | -4.576, 0.770 | -1.665 | -3.455, 0.124 | 0.83 |
| | Model 2 | 11 or younger | 1,364 | 88.20 | 15.30 | 2.752 | 1.060, 4.444 | 3.172 | 2.246, 4.099 | 0.67 |
| | | 12-13 | 3,981 | 84.48 | 13.80 | Ref | | Ref | | NA |
| | | 14-15 | 1,966 | 82.53 | 12.71 | -0.868 | -2.263, 0.528 | -2.755 | -3.578, -1.932 | 0.07 |
| | | 16 or higher | 335 | 83.66 | 14.49 | -1.902 | -4.569, 0.765 | -2.056 | -3.817, -0.294 | 0.98 |
| | Model 3 | 11 or younger | 1,166 | 87.64 | 14.97 | 2.598 | 0.809, 4.387 | 3.068 | 2.108, 4.027 | 0.72 |
| | | 12-13 | 3,437 | 84.05 | 13.46 | Ref | | Ref | | NA |
| | | 14-15 | 1,672 | 81.95 | 12.19 | -1.235 | -2.710, 0.239 | -2.642 | -3.496, -1.789 | 0.20 |
| | | 16 or higher | 282 | 83.02 | 13.90 | -3.069 | -5.657, -0.481 | -1.686 | -3.510, 0.137 | 0.49 |

CI=confidence interval; HDL=high-density lipoprotein; SD=standard deviation.

Model 1 Adjusted for age
Model 2 Adjusted for age, ethnicity, qualifications, parental history of cardiovascular disease and parental history of diabetes. Blood pressure further adjusted for use of antihypertensive drugs, cholesterol levels adjusted for lipid lowering drugs and glucose adjusted for use of antidiabetic drugs.
Model 3 Adjusted for age, ethnicity, qualifications, parental history of cardiovascular disease, parental history of diabetes, smoking, alcohol intake and leisure time physical activity. Blood pressure further adjusted for use of antihypertensive drugs, cholesterol levels adjusted for lipid lowering drugs and glucose adjusted for use of antidiabetic drugs.

*Outcome log transformed and the coefficients reflect the percent change in the outcome.

**Table S5.** Association Between Age at Menarche and 10-Year Risk For Overall Cardiovascular Disease, the Scottish Family Health Study, 2006-2011.

| Risk score | Model | Age at menarche | N | Mean | SD | Within-sibships association | | Between-sibships association | | Bootstrap p-value for the difference in the within and between sibship association |
|---|---|---|---|---|---|---|---|---|---|---|
| | | | | | | β | 95% CI | β | 95% CI | |
| Framingham risk score * | Model 1 | 11 or younger | 1,076 | 26.06 | 0.91 | 0.09 | 0.02, 0.17 | 0.10 | 0.06, 0.14 | 0.80 |
| | | 12-13 | 3,014 | 25.94 | 0.90 | Ref | | Ref | | NA |
| | | 14-15 | 1,543 | 25.97 | 0.92 | 0.02 | -0.04, 0.08 | -0.03 | -0.07, 0.00 | 0.24 |
| | | 16 or higher | 272 | 26.07 | 0.87 | -0.03 | -0.15, 0.09 | 0.04 | -0.04, 0.12 | 0.54 |
| | Model 2 | 11 or younger | 1,076 | 26.06 | 0.91 | 0.08 | 0.00, 0.16 | 0.09 | 0.05, 0.13 | 0.85 |
| | | 12-13 | 3,014 | 25.94 | 0.90 | Ref | | Ref | | NA |
| | | 14-15 | 1,543 | 25.97 | 0.92 | 0.02 | -0.04, 0.08 | -0.04 | -0.07, 0.00 | 0.20 |
| | | 16 or higher | 272 | 26.07 | 0.87 | -0.03 | -0.15, 0.09 | 0.02 | -0.05, 0.09 | 0.68 |
| | Model 3 | 11 or younger | 922 | 26.04 | 0.90 | 0.08 | -0.01, 0.17 | 0.08 | 0.04, 0.13 | 0.85 |
| | | 12-13 | 2,591 | 25.92 | 0.89 | Ref | | Ref | | NA |
| | | 14-15 | 1,312 | 25.94 | 0.91 | 0.02 | -0.05, 0.09 | -0.02 | -0.06, 0.01 | 0.40 |
| | | 16 or higher | 231 | 26.01 | 0.84 | -0.04 | -0.18, 0.10 | 0.01 | -0.07, 0.08) | 0.71 |
| NHANES ECG risk equation score † | Model 1 | 11 or younger | 1,076 | 7.963 | 0.847 | 0.030 | -0.004, 0.064 | 0.018 | 0.001, 0.035 | 0.62 |
| | | 12-13 | 3,054 | 7.928 | 0.845 | Ref | | Ref | | NA |
| | | 14-15 | 1,581 | 7.970 | 0.880 | 0.002 | -0.025, 0.030 | -0.020 | -0.035, -0.005 | 0.27 |
| | | 16 or higher | 265 | 8.061 | 0.819 | 0.003 | -0.056, 0.063 | 0.027 | -0.005, 0.059 | 0.82 |
| | Model 2 | 11 or younger | 1,076 | 7.963 | 0.847 | 0.031 | -0.003, 0.065 | 0.015 | -0.002, 0.032 | 0.52 |
| | | 12-13 | 3,054 | 7.928 | 0.845 | Ref | | Ref | | NA |
| | | 14-15 | 1,581 | 7.970 | 0.880 | 0.004 | -0.024, 0.031 | -0.020 | -0.035, -0.005 | 0.24 |
| | | 16 or higher | 265 | 8.061 | 0.819 | 0.003 | -0.057, 0.064 | 0.025 | -0.007, 0.057 | 0.85 |
| | Model 3 | 11 or younger | 919 | 7.957 | 0.846 | 0.044 | 0.005, 0.083 | 0.023 | 0.006, 0.041 | 0.43 |
| | | 12-13 | 2,625 | 7.914 | 0.845 | Ref | | Ref | | NA |
| | | 14-15 | 1,336 | 7.928 | 0.865 | -0.015 | -0.047, 0.017 | -0.014 | -0.030, 0.001 | 0.93 |
| | | 16 or higher | 224 | 8.025 | 0.783 | -0.001 | -0.073, 0.071 | 0.027 | -0.005, 0.060 | 0.79 |

CI=confidence interval; ECG=electrocardiogram; SD=standard deviation.

* The variables included in the Framingham risk score is age, total cholesterol, HDL cholesterol, systolic blood pressure, smoking and diabetes.

† The information included in the NHANES ECG risk score included age, positive deflection of T axis, negative deflection of the T axis, heart rate and corrected QT interval.

Model 1 Adjusted for age

Model 2 Adjusted for age, ethnicity, qualifications, parental history of cardiovascular disease and parental history of diabetes.

Model 3 Adjusted for all of the covariates in Model 2 in addition to smoking (not adjusted for in the analysis of the Framingham risk score since part of the risk calculation), alcohol intake and leisure time physical activity.

**Table S6.** Associations of Age at menarche With Blood pressure, Cholesterol and Glucose Excluding Individuals On Medications That Might Influence the Outcomes, the Scottish Family Health Study, 2006-2011.

| Outcome | Model | Age at menarche | N | Mean/Median | SD/Range | Within-sibships association | | Between-sibships association | | Bootstrap p-value for the difference in the within and between sibship association |
|---|---|---|---|---|---|---|---|---|---|---|
| | | | | | | β | 95% CI | β | 95% CI | |
| Systolic blood pressure (mmHg) | Model 1 | 11 or younger | 1,249 | 126.34 | 16.66 | 1.81 | -0.40, 4.02 | 2.21 | 1.19, 3.24 | 0.80 |
| | | 12-13 | 3,728 | 123.79 | 15.72 | Ref | | Ref | | NA |
| | | 14-15 | 1,853 | 124.56 | 16.80 | -0.07 | -1.79, 1.65 | -0.26 | -1.17, 0.64 | 0.81 |
| | | 16 or higher | 313 | 125.04 | 16.20 | -2.69 | -6.23, 0.85 | 0.03 | -1.89, 1.96 | 0.39 |
| | Model 2 | 11 or younger | 1,249 | 126.34 | 16.66 | 1.55 | -0.67, 3.77 | 2.00 | 0.98, 3.01 | 0.79 |
| | | 12-13 | 3,728 | 123.79 | 15.72 | Ref | | Ref | | NA |
| | | 14-15 | 1,853 | 124.56 | 16.80 | -0.08 | -1.79, 1.63 | -0.28 | -1.18, 0.62 | 0.83 |
| | | 16 or higher | 313 | 125.04 | 16.20 | -2.84 | -6.39, 0.70 | -0.18 | -2.09, 1.74 | 0.40 |
| | Model 3 | 11 or younger | 1,076 | 126.27 | 16.75 | 2.12 | -0.53, 4.77 | 2.05 | 0.98, 3.13 | 0.97 |
| | | 12-13 | 3,221 | 123.63 | 15.55 | Ref | | Ref | | NA |
| | | 14-15 | 1,580 | 124.13 | 16.55 | 0.50 | -1.44, 2.44 | -0.30 | -1.25, 0.65 | 0.56 |
| | | 16 or higher | 268 | 124.74 | 15.83 | -2.46 | -6.15, 1.23 | -0.46 | -2.47, 1.56 | 0.54 |
| Diastolic blood pressure (mmHg) | Model 1 | 11 or younger | 1,249 | 78.76 | 9.99 | 1.29 | -0.10, 2.67 | 1.43 | 0.77, 2.09 | 0.88 |
| | | 12-13 | 3,728 | 77.12 | 9.68 | Ref | | Ref | | NA |
| | | 14-15 | 1,853 | 76.82 | 10.0 | -0.49 | -1.57, 0.60 | -0.79 | -1.38, -0.21 | 0.67 |
| | | 16 or higher | 313 | 77.60 | 9.86 | -0.08 | -2.37, 2.21 | -0.39 | -1.64, 0.85 | 0.79 |
| | Model 2 | 11 or younger | 1,249 | 78.76 | 9.99 | 1.14 | -0.25, 2.53 | 1.30 | 0.65, 1.96 | 0.86 |
| | | 12-13 | 3,728 | 77.12 | 9.68 | Ref | | Ref | | NA |
| | | 14-15 | 1,853 | 76.82 | 10.0 | -0.49 | -1.57, 0.59 | -0.78 | -1.36, -0.20 | 0.70 |
| | | 16 or higher | 313 | 77.60 | 9.86 | -0.19 | -2.49, 2.11 | -0.43 | -1.66, 0.81 | 0.81 |
| | Model 3 | 11 or younger | 1,076 | 78.71 | 10.02 | 1.51 | -0.13, 3.15 | 1.34 | 0.64, 2.04 | 0.92 |
| | | 12-13 | 3,221 | 76.99 | 9.65 | Ref | | Ref | | NA |
| | | 14-15 | 1,580 | 76.72 | 10.05 | -0.20 | -1.41, 1.00 | -0.64 | -1.26, -0.01 | 0.57 |
| | | 16 or higher | 268 | 77.65 | 10.03 | -0.24 | -2.81, 2.33 | -0.11 | -1.42, 1.20 | 0.98 |
| HDL cholesterol, (mmol/L) | Model 1 | 11 or younger | 1,266 | 1.540 | 0.408 | -0.056 | -0.102, -0.010 | -0.050 | -0.079, -0.021 | 0.93 |
| | | 12-13 | 3,675 | 1.595 | 0.415 | Ref | | Ref | | NA |
| | | 14-15 | 1,806 | 1.626 | 0.404 | -0.013 | -0.056, 0.030 | 0.032 | 0.006, 0.058 | 0.18 |
| | | 16 or higher | 308 | 1.658 | 0.436 | -0.023 | -0.116, 0.070 | 0.055 | 0.000, 0.110 | 0.25 |
| | Model 2 | 11 or younger | 1,266 | 1.540 | 0.408 | -0.054 | -0.100, -0.008 | -0.042 | -0.071, -0.013 | 0.78 |
| | | 12-13 | 3,675 | 1.595 | 0.415 | Ref | | Ref | | NA |

| | | | | | | | | | | |
|---|---|---|---|---|---|---|---|---|---|---|
| | | 14-15 | 1,806 | 1.626 | 0.404 | -0.014 | -0.057, 0.030 | 0.034 | 0.008, 0.060 | 0.16 |
| | | 16 or higher | 308 | 1.658 | 0.436 | -0.022 | -0.115, 0.071 | 0.064 | 0.009, 0.118 | 0.21 |
| | Model 3 | 11 or younger | 1,101 | 1.551 | 0.410 | -0.034 | -0.087, 0.017 | -0.044 | -0.074, -0.014 | 0.77 |
| | | 12-13 | 3,173 | 1.608 | 0.412 | Ref | | Ref | | NA |
| | | 14-15 | 1,546 | 1.638 | 0.408 | -0.009 | -0.058, 0.040 | 0.029 | 0.003, 0.056 | 0.32 |
| | | 16 or higher | 265 | 1.679 | 0.442 | -0.009 | -0.103, 0.084 | 0.054 | -0.002, 0.111 | 0.33 |
| Non-HDL cholesterol (mmol/L) | Model 1 | 11 or younger | 1,266 | 3.657 | 1.070 | 0.210 | 0.087, 0.334 | 0.062 | -0.006, 0.129 | 0.17 |
| | | 12-13 | 3,675 | 3.530 | 1.050 | Ref | | Ref | | NA |
| | | 14-15 | 1,806 | 3.514 | 1.032 | -0.013 | -0.122, 0.095 | -0.097 | -0.157, -0.037 | 0.31 |
| | | 16 or higher | 308 | 3.574 | 1.001 | -0.077 | -0.270, 0.117 | -0.060 | -0.188, 0.067 | 0.97 |
| | Model 2 | 11 or younger | 1,266 | 3.657 | 1.070 | 0.209 | 0.085, 0.334 | 0.048 | -0.020, 0.115 | 0.13 |
| | | 12-13 | 3,675 | 3.530 | 1.050 | Ref | | Ref | | NA |
| | | 14-15 | 1,806 | 3.514 | 1.032 | -0.011 | -0.119, 0.097 | -0.101 | -0.161, -0.041 | 0.28 |
| | | 16 or higher | 308 | 3.574 | 1.001 | -0.086 | -0.280, 0.107 | -0.066 | -0.194, 0.061 | 0.95 |
| | Model 3 | 11 or younger | 1,101 | 3.641 | 1.076 | 0.142 | 0.006, 0.279 | 0.065 | -0.006, 0.135 | 0.49 |
| | | 12-13 | 3,173 | 3.503 | 1.044 | Ref | | Ref | | NA |
| | | 14-15 | 1,546 | 3.477 | 1.020 | -0.036 | -0.157, 0.086 | -0.084 | -0.147, -0.021 | 0.57 |
| | | 16 or higher | 265 | 3.536 | 0.989 | -0.109 | -0.321, 0.102 | -0.079 | -0.213, 0.054 | 0.94 |
| Glucose, (mmol/L)* | Model 1 | 11 or younger | 1,305 | 4.6 | 4.3, 4.9 | -0.71 | -2.21, 0.79 | 0.82 | 0.03, 1.61 | 0.20 |
| | | 12-13 | 3,780 | 4.5 | 4.3, 4.8 | Ref | | Ref | | NA |
| | | 14-15 | 1,864 | 4.6 | 4.3, 4.8 | 1.11 | -0.16, 2.37 | -0.54 | -1.25, 0.17 | 0.12 |
| | | 16 or higher | 324 | 4.6 | 4.3, 4.8 | -0.64 | -3.72, 2.43 | -1.08 | -2.59, 0.43 | 0.99 |
| | Model 2 | 11 or younger | 1,305 | 4.6 | 4.3, 4.9 | -0.73 | -2.22, 0.77 | 0.80 | 0.01, 1.59 | 0.18 |
| | | 12-13 | 3,780 | 4.5 | 4.3, 4.8 | Ref | | Ref | | NA |
| | | 14-15 | 1,864 | 4.6 | 4.3, 4.8 | 1.13 | -0.14, 2.40 | -0.54 | -1.25, 0.17 | 0.12 |
| | | 16 or higher | 324 | 4.6 | 4.3, 4.8 | -0.76 | -3.86, 2.34 | -1.01 | -2.52, 0.50 | 0.94 |
| | Model 3 | 11 or younger | 1,120 | 4.6 | 4.3, 4.9 | -0.47 | -2.20, 1.25 | 1.01 | 0.18, 1.83 | 0.25 |
| | | 12-13 | 3,255 | 4.5 | 4.3, 4.8 | Ref | | Ref | | NA |
| | | 14-15 | 1,593 | 4.6 | 4.3, 4.8 | 1.36 | -0.14, 2.86 | -0.37 | -1.11, 0.37 | 0.16 |
| | | 16 or higher | 274 | 4.6 | 4.3, 4.8 | -2.64 | -6.33, 1.04 | -0.55 | -2.13, 1.02 | 0.51 |

CI=confidence interval; HDL=high-density lipoprotein; SD=standard deviation.
Model 1 Adjusted for age
Model 2 Adjusted for age, ethnicity, qualifications, parental history of cardiovascular disease and parental history of diabetes.
Model 3 Adjusted for age, ethnicity, qualifications, parental history of cardiovascular disease, parental history of diabetes, smoking, alcohol intake and leisure time physical activity.
* Outcome log transformed and the coefficients reflect the percent change in the outcome.

**Table S7.** Associations of Age at Menarche With Blood Pressure, Cholesterol and Glucose After Adjustment for Body-Mass Index, the Scottish Family Health Study, 2006-2011.

| Outcome | Model | Age at menarche | N | Mean /Median | SD/Range | Within-sibships association | | Between-sibships association | | Bootstrap p-value for the difference in the within and between sibship association |
|---|---|---|---|---|---|---|---|---|---|---|
| | | | | | | β | 95% CI | β | 95% CI | |
| Systolic blood pressure (mmHg) | Model 1 | 11 or younger | 1,189 | 127.3 | 17.2 | 2.26 | -0.26, 4.78 | 1.74 | 0.68, 2.80 | 0.78 |
| | | 12-13 | 3,474 | 124.7 | 16.3 | Ref | | Ref | | NA |
| | | 14-15 | 1,687 | 125.1 | 17.3 | 0.44 | -1.45, 2.32 | -0.41 | -1.35, 0.53 | 0.54 |
| | | 16 or higher | 285 | 126.1 | 17.4 | -2.74 | -6.52, 1.04 | 0.31 | -1.69, 2.32 | 0.34 |
| | Model 2 | 11 or younger | 1,174 | 127.37 | 17.19 | 0.62 | -1.88, 3.12 | 0.55 | -0.48, 1.58 | 0.94 |
| | | 12-13 | 3,448 | 124.71 | 16.26 | Ref | | Ref | | NA |
| | | 14-15 | 1,674 | 125.14 | 17.32 | 1.10 | -0.71, 2.92 | 0.53 | -0.38, 1.45 | 0.65 |
| | | 16 or higher | 280 | 126.25 | 17.50 | -1.38 | -5.10, 2.34 | 1.05 | -0.90, 2.99 | 0.48 |
| Diastolic blood pressure (mmHg) | Model 1 | 11 or younger | 1,392 | 79.2 | 10.1 | 1.74 | 0.22, 3.25 | 1.39 | 0.71, 2.08 | 0.75 |
| | | 12-13 | 4,034 | 77.5 | 9.9 | Ref | | Ref | | NA |
| | | 14-15 | 1,989 | 77.1 | 10.0 | -0.37 | -1.52, 0.78 | -0.66 | -1.27, -0.05 | 0.67 |
| | | 16 or higher | 339 | 77.8 | 10.1 | -0.93 | -3.40, 1.54 | -0.06 | -1.36, 1.24 | 0.74 |
| | Model 2 | 11 or younger | 1,174 | 79.2 | 10.0 | 0.52 | -0.96, 1.99 | 0.34 | -0.30, 0.99 | 0.84 |
| | | 12-13 | 3,448 | 77.4 | 9.8 | Ref | | Ref | | NA |
| | | 14-15 | 1,674 | 77.0 | 10.1 | 0.14 | -0.96, 1.23 | 0.14 | -0.43, 0.72 | 0.91 |
| | | 16 or higher | 280 | 78.0 | 10.2 | 0.55 | -1.76, 2.86 | 0.58 | -0.64, 1.80 | 0.87 |
| HDL cholesterol, (mmol/L) | Model 1 | 11 or younger | 1,338 | 1.537 | 0.404 | -0.042 | -0.093, 0.008 | -0.038 | -0.067, -0.009 | 0.96 |
| | | 12-13 | 3,851 | 1.591 | 0.418 | Ref | | Ref | | NA |
| | | 14-15 | 1,897 | 1.627 | 0.405 | -0.009 | -0.057, 0.039 | 0.037 | 0.011, 0.063 | 0.22 |
| | | 16 or higher | 326 | 1.646 | 0.432 | -0.017 | -0.105, 0.071 | 0.061 | 0.005, 0.116 | 0.24 |
| | Model 2 | 11 or younger | 1,132 | 1.549 | 0.404 | -0.005 | -0.054, 0.045 | 0.002 | -0.026, 0.030 | 0.87 |
| | | 12-13 | 3,292 | 1.605 | 0.415 | Ref | | Ref | | NA |
| | | 14-15 | 1,602 | 1.639 | 0.408 | -0.026 | -0.071, 0.020 | 0.007 | -0.018, 0.032 | 0.38 |
| | | 16 or higher | 271 | 1.673 | 0.438 | -0.037 | -0.126, 0.052 | 0.030 | -0.023, 0.083 | 0.30 |
| Non-HDL cholesterol (mmol/L) | Model 1 | 11 or younger | 1,338 | 3.638 | 1.065 | 0.142 | 0.009, 0.274 | 0.068 | -0.002, 0.137 | 0.49 |
| | | 12-13 | 3,851 | 3.517 | 1.050 | Ref | | Ref | | NA |
| | | 14-15 | 1,897 | 3.498 | 1.031 | -0.013 | -0.132, 0.105 | -0.092 | -0.154, -0.030 | 0.37 |
| | | 16 or higher | 326 | 3.583 | 1.008 | 0.071 | -0.281, 0.139 | -0.070 | -0.201, 0.061 | 0.96 |
| | Model 2 | 11 or younger | 1,132 | 3.631 | 1.067 | 0.038 | -0.091, 0.167 | -0.006 | -0.074, 0.062 | 0.68 |
| | | 12-13 | 3,292 | 3.491 | 1.039 | Ref | | Ref | | NA |

| | | | | | | | | | |
|---|---|---|---|---|---|---|---|---|---|
| | | 14-15 | 1,602 | 3.460 | 1.023 | 0.010 | -0.106, 0.126 | -0.041 | -0.102, 0.020 | 0.55 |
| | | 16 or higher | 271 | 3.544 | 1.005 | 0.005 | -0.202, 0.212 | -0.028 | -0.157, 0.100 | 0.78 |
| Glucose, (mmol/L)* | Model 1 | 11 or younger | 1,317 | 4.6 | 4.3, 4.9 | -0.022 | -2.002, 1.958 | 1.006 | 0.125, 1.888 | 0.43 |
| | | 12-13 | 3,814 | 4.5 | 4.3, 4.8 | Ref | | Ref | | NA |
| | | 14-15 | 1,876 | 4.6 | 4.3, 4.8 | 1.534 | 0.021, 3.047 | -0.212 | -0.999, 0.576 | 0.17 |
| | | 16 or higher | 327 | 4.6 | 4.3, 4.8 | -2.585 | -6.208, 1.038 | -0.721 | -2.396, 0.953 | 0.54 |
| | Model 2 | 11 or younger | 1,112 | 4.6 | 4.3, 4.9 | -1.125 | -3.057, 0.808 | 0.516 | -0.372, 1.403 | 0.26 |
| | | 12-13 | 3,259 | 4.5 | 4.3, 4.8 | Ref | | Ref | | NA |
| | | 14-15 | 1,586 | 4.6 | 4.3, 4.8 | 1.500 | 0.047, 2.953 | 0.108 | -0.681, 0.897 | 0.27 |
| | | 16 or higher | 271 | 4.6 | 4.3, 4.8 | -2.023 | -5.570, 1.524 | -0.230 | -1.902, 1.443 | 0.58 |

CI=confidence interval; HDL=high-density lipoprotein; SD=standard deviation.

Model 1 Adjusted for age, ethnicity, qualifications, parental history of cardiovascular disease, parental history of diabetes, smoking, alcohol intake and leisure time physical activity. Blood pressure further adjusted for use of antihypertensive drugs, cholesterol levels adjusted for lipid lowering drugs and glucose adjusted for use of antidiabetic drugs (Model 3 from Supplement Table 4).

Model 2 Adjusted for all covariates in model 1 in addition to adult body-mass index.

*Outcomes log transformed and the coefficients reflect the percent change in the outcome.

**Table S8.** Association Between Age at Menarche and 10-Year Risk for Cardiovascular Disease After Adjustment for Adult Body-Mass Index, the Scottish Family Health Study, 2006-2011.

| Risk score | Model | Age at menarche | N | Mean | SD | Within-sibships association | | Between-sibships association | | Bootstrap p-value for the difference in the within and between sibship association |
|---|---|---|---|---|---|---|---|---|---|---|
| | | | | | | β | 95% CI | β | 95% CI | |
| Framingham risk score * | Model 1 | 11 or younger | 922 | 26.04 | 0.90 | 0.08 | -0.01, 0.17 | 0.08 | 0.04, 0.13 | 0.85 |
| | | 12-13 | 2,591 | 25.92 | 0.89 | Ref | | Ref | | NA |
| | | 14-15 | 1,312 | 25.94 | 0.91 | 0.02 | -0.05, 0.09 | -0.02 | -0.06, 0.01 | 0.40 |
| | | 16 or higher | 231 | 26.01 | 0.84 | -0.04 | -0.18, 0.10 | 0.01 | -0.07, 0.08 | 0.71 |
| | Model 2 | 11 or younger | 913 | 26.04 | 0.90 | 0.00 | -0.08, 0.09 | 0.02 | -0.02, 0.06 | 0.78 |
| | | 12-13 | 2,575 | 25.92 | 0.89 | Ref | | Ref | | NA |
| | | 14-15 | 1,304 | 25.94 | 0.91 | 0.04 | -0.02, 0.11 | 0.02 | -0.01, 0.06 | 0.67 |
| | | 16 or higher | 227 | 26.02 | 0.84 | 0.02 | -0.11, 0.14 | 0.04 | -0.03, 0.11 | 0.91 |
| NHANES ECG risk equation † | Model 1 | 11 or younger | 919 | 7.957 | 0.846 | 0.044 | 0.005, 0.083 | 0.023 | 0.006, 0.041 | 0.43 |
| | | 12-13 | 2,625 | 7.914 | 0.845 | Ref | | Ref | | NA |
| | | 14-15 | 1,336 | 7.928 | 0.865 | -0.015 | -0.047, 0.017 | -0.014 | -0.030, 0.001 | 0.93 |
| | | 16 or higher | 224 | 8.025 | 0.783 | -0.001 | -0.073, 0.071 | 0.027 | -0.005, 0.060 | 0.79 |
| | Model 2 | 11 or younger | 909 | 7.961 | 0.848 | 0.022 | -0.016, 0.059 | 0.004 | -0.014, 0.021 | 0.45 |
| | | 12-13 | 2,607 | 7.913 | 0.844 | Ref | | Ref | | NA |
| | | 14-15 | 1,328 | 7.929 | 0.864 | -0.006 | -0.037, 0.026 | 0.000 | -0.015, 0.015 | 0.94 |
| | | 16 or higher | 220 | 8.029 | 0.786 | 0.005 | -0.064, 0.074 | 0.034 | 0.002, 0.065 | 0.75 |

CI=confidence interval; ECG=electrocardiogram; SD=standard deviation.

* The variables included in the Framingham risk score is age, total cholesterol, HDL cholesterol, systolic blood pressure, smoking and diabetes.
† The information included in the NHANES ECG risk score included age, positive deflection of T axis, negative deflection of the T axis, heart rate and corrected QT interval.
Model 1 Adjusted for age, ethnicity, qualifications, parental history of cardiovascular disease, parental history of diabetes, smoking, alcohol intake and leisure time physical activity (Model 3 from Table 2).
Model 2 Adjusted for all covariates in model 1 in addition to adult body-mass index.

**Table S9.** Association Between Age at Menarche and Cardiometabolic Health After Restricting the Analysis to Sibships With up to Four Years Age Difference Between Sisters, the Scottish Family Health Study, 2006-2011.

| Outcome | Model | Age at menarche | N | Mean/Median | SD/Range | Within-sibships association | | Between-sibships association | | Bootstrap p-value for the difference in the within and between sibship association |
|---|---|---|---|---|---|---|---|---|---|---|
| | | | | | | β | 95% CI | β | 95% CI | |
| Systolic blood pressure (mmHg) | Model 1 | 11 or younger | 1,276 | 127.4 | 17.1 | 2.67 | 0.14, 5.20 | 1.87 | 0.84, 2.90 | 0.69 |
| | | 12-13 | 3,757 | 124.9 | 16.6 | Ref | | Ref | | NA |
| | | 14-15 | 1,855 | 125.9 | 17.8 | 1.20 | -0.76, 3.15 | -0.26 | -1.17, 0.65 | 0.25 |
| | | 16 or higher | 313 | 126.6 | 18.4 | -2.46 | -6.58, 1.67 | 0.28 | -1.67, 2.22 | 0.41 |
| | Model 2 | 11 or younger | 1,276 | 127.4 | 17.1 | 2.35 | -0.19, 4.88 | 1.53 | 0.52, 2.55 | 0.70 |
| | | 12-13 | 3,757 | 124.9 | 16.6 | Ref | | Ref | | NA |
| | | 14-15 | 1,855 | 125.9 | 17.8 | 1.24 | -0.71, 3.20 | -0.20 | -1.09, 0.70 | 0.27 |
| | | 16 or higher | 313 | 126.6 | 18.4 | -2.50 | -6.65, 1.66 | 0.26 | -1.66, 2.18 | 0.41 |
| | Model 3 | 11 or younger | 1,090 | 127.2 | 17.1 | 3.01 | 0.09, 5.93 | 1.60 | 0.53, 2.67 | 0.58 |
| | | 12-13 | 3,235 | 124.7 | 16.3 | Ref | | Ref | | NA |
| | | 14-15 | 1,569 | 125.4 | 17.5 | 1.75 | -0.45, 3.93 | -0.24 | -1.20, 0.71 | 0.19 |
| | | 16 or higher | 266 | 126.2 | 17.8 | 1.74 | -0.45, 3.93 | 0.05 | -1.98, 2.08 | 0.29 |
| Diastolic blood pressure (mmHg) | Model 1 | 11 or younger | 1,276 | 79.2 | 10.0 | 1.53 | 0.03, 3.03 | 1.38 | 0.72, 2.03 | 0.89 |
| | | 12-13 | 3,757 | 77.5 | 9.9 | Ref | | Ref | | NA |
| | | 14-15 | 1,855 | 77.1 | 10.1 | 0.06 | -1.15, 1.26 | -0.77 | -1.34, -0.19 | 0.27 |
| | | 16 or higher | 313 | 77.6 | 10.2 | -0.27 | -2.94, 2.40 | -0.56 | -1.79, 0.68 | 0.79 |
| | Model 2 | 11 or younger | 1,276 | 79.2 | 10.0 | 1.30 | -0.19, 2.79 | 1.22 | 0.57, 1.87 | 0.95 |
| | | 12-13 | 3,757 | 77.5 | 9.9 | Ref | | Ref | | NA |
| | | 14-15 | 1,855 | 77.1 | 10.1 | 0.07 | -1.13, 1.28 | -0.74 | -1.32, -0.17 | 0.27 |
| | | 16 or higher | 313 | 77.6 | 10.2 | -0.30 | -2.98, 2.37 | -0.55 | -1.78, 0.68 | 0.80 |
| | Model 3 | 11 or younger | 1,090 | 79.1 | 10.1 | 1.93 | 0.21, 3.66 | 1.31 | 0.62, 2.00 | 0.67 |
| | | 12-13 | 3,235 | 77.3 | 9.9 | Ref | | Ref | | NA |
| | | 14-15 | 1,569 | 77.0 | 10.2 | 0.30 | -1.03, 1.63 | -0.63 | -1.24, -0.01 | 0.27 |
| | | 16 or higher | 266 | 77.7 | 10.5 | -1.08 | -4.10, 1.93 | -0.18 | -1.49, 1.14 | 0.75 |
| HDL cholesterol, (mmol/L) | Model 1 | 11 or younger | 1,228 | 1.534 | 0.405 | -0.061 | -0.114, -0.008 | -0.049 | -0.078, -0.020 | 0.81 |
| | | 12-13 | 3,591 | 1.589 | 0.414 | Ref | | Ref | | NA |
| | | 14-15 | 1,768 | 1.625 | 0.406 | -0.022 | -0.069, 0.026 | 0.038 | 0.013, 0.064 | 0.08 |
| | | 16 or higher | 300 | 1.661 | 0.436 | -0.037 | -0.139, 0.061 | 0.073 | 0.018, 0.127 | 0.14 |
| | Model 2 | 11 or younger | 1,228 | 1.534 | 0.405 | -0.061 | -0.114, -0.007 | -0.042 | -0.070, -0.014 | 0.68 |
| | | 12-13 | 3,591 | 1.589 | 0.414 | Ref | | Ref | | NA |
| | | 14-15 | 1,768 | 1.625 | 0.406 | -0.02 | -0.071, 0.024 | 0.039 | 0.014, 0.065 | 0.07 |

| | | | | | | | | | | |
|---|---|---|---|---|---|---|---|---|---|---|
| | | 16 or higher | 300 | 1.661 | 0.436 | -0.036 | -0.137, 0.065 | 0.083 | 0.029, 0.137 | 0.12 |
| | Model 3 | 11 or younger | 1,053 | 1.546 | 0.406 | -0.036 | -0.097, 0.025 | -0.040 | -0.069, -0.010 | 0.88 |
| | | 12-13 | 3,089 | 1.603 | 0.410 | Ref | | Ref | | NA |
| | | 14-15 | 1,500 | 1.638 | 0.409 | -0.022 | -0.076, 0.032 | 0.035 | 0.008, 0.061 | 0.15 |
| | | 16 or higher | 257 | 1.684 | 0.441 | -0.024 | -0.122, 0.074 | 0.073 | 0.017, 0.128 | 0.20 |
| Non-HDL cholesterol (mmol/L) | Model 1 | 11 or younger | 1,228 | 3.630 | 1.056 | 0.159 | 0.009, 0.309 | 0.070 | 0.002, 0.138 | 0.49 |
| | | 12-13 | 3,591 | 3.518 | 1.057 | Ref | | Ref | | NA |
| | | 14-15 | 1,768 | 3.491 | 1.025 | -0.006 | -0.132, 0.120 | -0.089 | -0.149, -0.028 | 0.27 |
| | | 16 or higher | 300 | 3.568 | 0.996 | -0.072 | -0.295, 0.151 | -0.049 | -0.178, 0.080 | 0.99 |
| | Model 2 | 11 or younger | 1,228 | 3.630 | 1.056 | 0.154 | 0.009, 0.299 | 0.055 | -0.012, 0.122 | 0.42 |
| | | 12-13 | 3,591 | 3.518 | 1.057 | Ref | | Ref | | NA |
| | | 14-15 | 1,768 | 3.491 | 1.025 | -0.008 | -0.130, 0.114 | -0.098 | -0.157, -0.039 | 0.24 |
| | | 16 or higher | 300 | 3.568 | 0.996 | -0.067 | -0.273, 0.139 | -0.067 | -0.193, 0.059 | 0.94 |
| | Model 3 | 11 or younger | 1,053 | 3.619 | 1.059 | 0.115 | -0.046, 0.276 | 0.059 | -0.011, 0.129 | 0.70 |
| | | 12-13 | 3,089 | 3.490 | 1.044 | Ref | | Ref | | NA |
| | | 14-15 | 1,500 | 3.452 | 1.013 | -0.031 | -0.166, 0.104 | -0.088 | -0.150, -0.026 | 0.49 |
| | | 16 or higher | 257 | 3.525 | 0.979 | -0.082 | -0.314, 0.151 | -0.100 | -0.232, 0.033 | 0.85 |
| Glucose, (mmol/L)* | Model 1 | 11 or younger | 1,207 | 4.6 | 4.3, 4.9 | -0.62 | -2.49, 1.24 | 1.24 | 0.34, 2.15 | 0.19 |
| | | 12-13 | 3,555 | 4.5 | 4.3, 4.8 | Ref | | Ref | | NA |
| | | 14-15 | 1,747 | 4.6 | 4.3, 4.8 | -0.22 | -1.83, 1.40 | -0.20 | -1.01, 0.60 | 0.94 |
| | | 16 or higher | 301 | 4.6 | 4.3, 4.8 | -3.06 | -6.54, 0.43 | -0.59 | -2.30, 1.13 | 0.32 |
| | Model 2 | 11 or younger | 1,207 | 4.6 | 4.3, 4.9 | -0.51 | -2.32, 1.30 | 1.10 | 0.24, 1.97 | 0.22 |
| | | 12-13 | 3,555 | 4.5 | 4.3, 4.8 | Ref | | Ref | | NA |
| | | 14-15 | 1,747 | 4.6 | 4.3, 4.8 | 0.02 | -1.53, 1.56 | -0.12 | -0.88, 0.65 | 0.98 |
| | | 16 or higher | 301 | 4.6 | 4.3, 4.8 | -3.08 | -6.61, 0.45 | -0.59 | -2.23, 1.05 | 0.29 |
| | Model 3 | 11 or younger | 1,033 | 4.6 | 4.3, 4.8 | 0.02 | -2.02, 2.05 | 0.94 | 0.03, 1.84 | 0.50 |
| | | 12-13 | 3,057 | 4.5 | 4.3, 4.9 | Ref | | Ref | | NA |
| | | 14-15 | 1,484 | 4.6 | 4.3, 4.8 | 0.84 | -0.86, 2.53 | -0.16 | -0.97, 0.65 | 0.48 |
| | | 16 or higher | 257 | 4.6 | 4.3, 4.8 | -4.14 | -8.69, 0.42 | -0.57 | -2.28, 1.14 | 0.32 |
| Body-mass index ($kg/m^2$) | Model 1 | 11 or younger | 1,260 | 28.0 | 6.1 | 1.70 | 0.87, 2.52 | 1.88 | 1.52, 2.25 | 0.73 |
| | | 12-13 | 3,721 | 26.1 | 5.2 | Ref | | Ref | | NA |
| | | 14-15 | 1,842 | 25.1 | 4.8 | -0.43 | -1.02, 0.17 | -1.24 | -1.56, -0.92 | 0.05 |
| | | 16 or higher | 309 | 25.3 | 5.3 | -0.86 | -2.05, 0.34 | -1.15 | -1.84, -0.47 | 0.87 |
| | Model 2 | 11 or younger | 1,260 | 28.0 | 6.1 | 1.60 | 0.78, 2.42 | 1.76 | 1.40, 2.11 | 0.77 |
| | | 12-13 | 3,721 | 26.1 | 5.2 | Ref | | Ref | | NA |
| | | 14-15 | 1,842 | 25.1 | 4.8 | -0.42 | -1.02, 0.18 | -1.24 | -1.55, -0.93 | 0.05 |
| | | 16 or higher | 309 | 25.3 | 5.3 | -0.87 | -2.05, 0.32 | -1.29 | -1.96, -0.62 | 0.75 |

| | | | | | | | | | | |
|---|---|---|---|---|---|---|---|---|---|---|
| | Model 3 | 11 or younger | 1,078 | 27.8 | 6.0 | 1.95 | 1.09, 2.82 | 1.68 | 1.31, 2.05 | 0.70 |
| | | 12-13 | 3,213 | 25.9 | 5.0 | Ref | | Ref | | NA |
| | | 14-15 | 1,559 | 24.9 | 4.6 | -0.75 | -1.39, -0.12 | -1.12 | -1.45, -0.79 | 0.43 |
| | | 16 or higher | 263 | 25.0 | 5.2 | -1.73 | -2.97, -0.49 | -1.13 | -1.83, -0.44 | 0.45 |
| Waist circumference, (cm) | Model 1 | 11 or younger | 1,249 | 88.1 | 15.2 | 3.07 | 0.98, 5.18 | 3.66 | 2.71, 4.60 | 0.67 |
| | | 12-13 | 3,707 | 84.4 | 13.8 | Ref | | Ref | | NA |
| | | 14-15 | 1,833 | 82.5 | 12.8 | -0.64 | -2.37, 1.09 | -2.53 | -3.36, -1.70 | 0.13 |
| | | 16 or higher | 309 | 83.2 | 14.1 | -2.67 | -5.75, 0.40 | -1.87 | -3.64, -0.09 | 0.71 |
| | Model 2 | 11 or younger | 1,249 | 88.1 | 15.2 | 2.89 | 0.79, 4.99 | 3.30 | 2.37, 4.23 | 0.76 |
| | | 12-13 | 3,707 | 84.4 | 13.8 | Ref | | Ref | | NA |
| | | 14-15 | 1,833 | 82.5 | 12.8 | -0.67 | -2.41, 1.06 | -2.58 | -3.40, -1.77 | 0.13 |
| | | 16 or higher | 309 | 83.2 | 14.1 | -2.65 | -5.70, 0.40 | -2.26 | -4.01, -0.51 | 0.84 |
| | Model 3 | 11 or younger | 1,067 | 87.7 | 14.9 | 3.30 | 1.15, 5.45 | 3.16 | 2.19, 4.13 | 0.92 |
| | | 12-13 | 3,200 | 83.9 | 13.4 | Ref | | Ref | | NA |
| | | 14-15 | 1,555 | 82.0 | 12.3 | -1.23 | -2.95, 0.50 | -2.43 | -3.29, -1.58 | 0.33 |
| | | 16 or higher | 263 | 82.5 | 13.5 | -3.72 | -6.63, -0.81 | -2.18 | -4.00, -0.35 | 0.48 |

CI=confidence interval; HDL=high-density lipoprotein; SD=standard deviation.
Model 1 Adjusted for age
Model 2 Adjusted for age, ethnicity, qualifications, parental history of cardiovascular disease and parental history of diabetes. Blood pressure further adjusted for use of antihypertensive drugs, cholesterol levels adjusted for lipid lowering drugs and glucose adjusted for use of antidiabetic drugs.
Model 3 Adjusted for all the characteristics in Model 2 in addition to smoking, alcohol intake and leisure time physical activity.
[*] Outcome log transformed and the coefficients reflect the percent change in the outcome.

**Table S10.** Association Between Age at Menarche and 10-Year Risk of Cardiovascular Disease After Restricting the Analysis to Sibships With up to Four Years Age Difference Between Sisters, the Scottish Family Health Study, 2006-2011

| Risk score | Model | Age at menarche | N | Mean | SD | Within-sibships association | | Between-sibships association | | Bootstrap p-value for the difference in the within and between sibship association |
|---|---|---|---|---|---|---|---|---|---|---|
| | | | | | | β | 95% CI | β | 95% CI | |
| Framingham risk score * | Model 1 | 11 or younger | 982 | 26.06 | 0.90 | 0.13 | 0.05, 0.22 | 0.09 | 0.05, 0.13 | 0.67 |
| | | 12-13 | 2,795 | 25.95 | 0.90 | Ref | | Ref | | NA |
| | | 14-15 | 1,430 | 25.98 | 0.93 | 0.04 | -0.03, 0.11 | -0.03 | -0.06, 0.01 | 0.21 |
| | | 16 or higher | 248 | 26.07 | 0.90 | -0.08 | -0.21, 0.05 | 0.03 | -0.05, 0.11 | 0.28 |
| | Model 2 | 11 or younger | 982 | 26.06 | 0.90 | 0.12 | 0.03, 0.21 | 0.08 | 0.04, 0.12 | 0.66 |
| | | 12-13 | 2,795 | 25.95 | 0.90 | Ref | | Ref | | NA |
| | | 14-15 | 1,430 | 25.98 | 0.93 | 0.04 | -0.03, 0.11 | -0.03 | -0.06, 0.01 | 0.18 |
| | | 16 or higher | 248 | 26.07 | 0.90 | -0.07 | -0.21, 0.06 | 0.01 | -0.06, 0.09 | 0.42 |
| | Model 3 | 11 or younger | 843 | 26.04 | 0.90 | 0.12 | 0.02, 0.22 | 0.08 | 0.03, 0.12 | 0.72 |
| | | 12-13 | 2,400 | 25.92 | 0.90 | Ref | | Ref | | NA |
| | | 14-15 | 1,211 | 25.95 | 0.92 | 0.04 | -0.03, 0.12 | -0.02 | -0.06, 0.02 | 0.27 |
| | | 16 or higher | 214 | 26.02 | 0.86 | -0.08 | -0.23, 0.07 | 0.00 | -0.08, 0.08 | 0.47 |
| NHANES ECG risk equation † | Model 1 | 11 or younger | 981 | 7.97 | 0.86 | 0.04 | 0.00, 0.08 | 0.02 | 0.00, 0.04 | 0.41 |
| | | 12-13 | 2,830 | 7.93 | 0.85 | Ref | | Ref | | NA |
| | | 14-15 | 1,464 | 7.98 | 0.89 | -0.01 | -0.04, 0.02 | -0.01 | -0.03, 0.00 | 0.84 |
| | | 16 or higher | 243 | 8.08 | 0.83 | -0.02 | -0.09, 0.06 | 0.03 | 0.00, 0.06 | 0.49 |
| | Model 2 | 11 or younger | 981 | 7.97 | 0.86 | 0.04 | 0.00, 0.08 | 0.02 | 0.00, 0.03 | 0.35 |
| | | 12-13 | 2,830 | 7.93 | 0.85 | Ref | | Ref | | NA |
| | | 14-15 | 1,464 | 7.98 | 0.89 | -0.01 | -0.04, 0.02 | -0.01 | -0.03, 0.00 | 0.77 |
| | | 16 or higher | 243 | 8.08 | 0.83 | -0.02 | -0.09, 0.06 | 0.03 | 0.00, 0.06 | 0.54 |
| | Model 3 | 11 or younger | 838 | 7.97 | 0.86 | 0.05 | 0.01, 0.10 | 0.02 | 0.01, 0.04 | 0.36 |
| | | 12-13 | 2,433 | 7.92 | 0.85 | Ref | | Ref | | NA |
| | | 14-15 | 1,233 | 7.94 | 0.87 | -0.03 | -0.07, 0.01 | -0.01 | -0.03, 0.00 | 0.63 |
| | | 16 or higher | 208 | 8.03 | 0.80 | -0.02 | -0.11, 0.07 | 0.03 | -0.01, 0.06 | 0.53 |

CI=confidence interval; ECG=electrocardiogram; SD=standard deviation.

* The variables included in the Framingham risk score is age, total cholesterol, HDL cholesterol, systolic blood pressure, smoking and diabetes.

† The information included in the NHANES ECG risk score included age, positive deflection of T axis, negative deflection of the T axis, heart rate and corrected QT interval.

The estimates are from a mixed effects linear regression analysis.

Model 1 Adjusted for age

Model 2 Adjusted for age, ethnicity, qualifications, parental history of cardiovascular disease and parental history of diabetes.

Model 3 Adjusted for all of the covariates in Model 2 in addition to smoking (not adjusted for in the analysis of the NHANES risk score since part of the risk calculation), alcohol intake and leisure time physical activity.

**Table S11.** Association Between Age at Menarche and Cardiometabolic Health Outcomes Excluding Individuals of Non-European Ethnicity, the Scottish Family Health Study, 2006-2011.

| Outcome | Model | Age at menarche | N | Mean/Median | SD/Range | Within-sibships association | | Between-sibships association | | Bootstrap p-value for the difference in the within and between sibship association |
|---|---|---|---|---|---|---|---|---|---|---|
| | | | | | | β | 95% CI | β | 95% CI | |
| Systolic blood pressure (mmHg) | Model 1 | 11 or younger | 1,362 | 127.7 | 17.2 | 1.95 | -0.19, 4.08 | 2.15 | 1.12, 3.18 | 0.98 |
| | | 12-13 | 3,959 | 124.9 | 16.5 | Ref | | Ref | | NA |
| | | 14-15 | 1,943 | 125.7 | 17.6 | -0.05 | -1.71, 1.61 | -0.45 | -1.36, 0.47 | 0.69 |
| | | 16 or higher | 334 | 126.6 | 18.0 | -2.56 | -6.09, 0.96 | 0.87 | -1.08, 2.82 | 0.33 |
| | Model 2 | 11 or younger | 1,362 | 127.7 | 17.2 | 1.82 | -0.33, 3.97 | 1.82 | 0.75, 2.89 | 0.91 |
| | | 12-13 | 3,959 | 124.9 | 16.5 | Ref | | Ref | | NA |
| | | 14-15 | 1,943 | 125.7 | 17.6 | -0.03 | -1.70, 1.64 | -0.34 | -1.29, 0.62 | 0.75 |
| | | 16 or higher | 334 | 126.6 | 18.0 | -2.63 | -6.16, 0.90 | 0.45 | -1.57, 2.48 | 0.32 |
| | Model 3 | 11 or younger | 1,165 | 127.4 | 17.3 | 2.35 | -0.19, 4.88 | 1.74 | 0.68, 2.80 | 0.74 |
| | | 12-13 | 3,408 | 124.7 | 16.3 | Ref | | Ref | | NA |
| | | 14-15 | 1,649 | 125.2 | 17.3 | 0.35 | -1.57, 2.26 | -0.41 | -1.35, 0.53 | 0.58 |
| | | 16 or higher | 281 | 126.2 | 17.4 | -3.02 | -6.85, 0.81 | 0.31 | -1.69, 2.32 | 0.29 |
| Diastolic blood pressure (mmHg) | Model 1 | 11 or younger | 1,362 | 79.2 | 10.1 | 1.42 | 0.14, 2.70 | 1.49 | 0.84, 2.15 | 0.98 |
| | | 12-13 | 3,959 | 77.5 | 9.9 | Ref | | Ref | | NA |
| | | 14-15 | 1,943 | 77.1 | 10.0 | -0.62 | -1.64, 0.41 | -0.84 | -1.43, -0.26 | 0.69 |
| | | 16 or higher | 334 | 77.8 | 10.1 | -0.41 | -2.55, 1.73 | -0.22 | -1.47, 1.02 | 0.97 |
| | Model 2 | 11 or younger | 1,362 | 79.2 | 10.1 | 1.38 | 0.09, 2.66 | 1.33 | 0.69, 1.98 | 0.89 |
| | | 12-13 | 3,959 | 77.5 | 9.9 | Ref | | Ref | | NA |
| | | 14-15 | 1,943 | 77.1 | 10.0 | -0.62 | -1.64, 0.41 | -0.80 | -1.38, -0.22 | 0.74 |
| | | 16 or higher | 334 | 77.8 | 10.1 | -0.45 | -2.61, 1.70 | -0.20 | -1.44, 1.03 | 0.99 |
| | Model 3 | 11 or younger | 1,165 | 79.2 | 10.1 | 1.81 | 0.29, 3.33 | 1.41 | 0.72, 2.10 | 0.67 |
| | | 12-13 | 3,408 | 77.4 | 9.8 | Ref | | Ref | | NA |
| | | 14-15 | 1,649 | 77.0 | 10.1 | -0.47 | -1.63, 0.69 | -0.60 | -1.21, 0.02 | 0.77 |
| | | 16 or higher | 281 | 77.9 | 10.3 | -1.02 | -3.52, 1.48 | -0.09 | -1.22, 1.40 | 0.65 |
| HDL cholesterol, (mmol/L) | Model 1 | 11 or younger | 1,309 | 1.538 | 0.405 | -0.065 | -0.110, -0.019 | -0.045 | -0.074, -0.016 | 0.62 |
| | | 12-13 | 3,779 | 1.592 | 0.418 | Ref | | Ref | | NA |
| | | 14-15 | 1,854 | 1.631 | 0.402 | -0.009 | -0.051, 0.033 | 0.044 | 0.018, 0.070 | 0.10 |
| | | 16 or higher | 321 | 1.645 | 0.433 | -0.032 | -0.118, 0.053 | 0.059 | 0.004, 0.114 | 0.18 |

| | | | | | | | | | | |
|---|---|---|---|---|---|---|---|---|---|---|
| | Model 2 | 11 or younger | 1,309 | 1.538 | 0.405 | -0.061 | -0.106, -0.016 | -0.037 | -0.066, -0.009 | 0.53 |
| | | 12-13 | 3,779 | 1.592 | 0.418 | Ref | | Ref | | NA |
| | | 14-15 | 1,854 | 1.631 | 0.402 | -0.010 | -0.052, 0.032 | 0.045 | 0.019, 0.070 | 0.10 |
| | | 16 or higher | 321 | 1.645 | 0.433 | -0.032 | -0.118, 0.053 | 0.067 | 0.013, 0.120 | 0.14 |
| | Model 3 | 11 or younger | 1,122 | 1.551 | 0.405 | -0.042 | -0.094, 0.009 | -0.038 | -0.067, -0.008 | 0.93 |
| | | 12-13 | 3,251 | 1.605 | 0.415 | Ref | | Ref | | NA |
| | | 14-15 | 1,580 | 1.644 | 0.405 | -0.011 | -0.059, 0.038 | 0.040 | 0.014, 0.066 | 0.18 |
| | | 16 or higher | 272 | 1.674 | 0.440 | -0.028 | -0.116, 0.061 | 0.062 | 0.006, 0.117 | 0.20 |
| Non-HDL cholesterol (mmol/L) | Model 1 | 11 or younger | 1,309 | 3.636 | 1.064 | 0.209 | 0.086, 0.332 | 0.065 | -0.003, 0.132 | 0.17 |
| | | 12-13 | 3,779 | 3.509 | 1.046 | Ref | | Ref | | NA |
| | | 14-15 | 1,854 | 3.496 | 1.031 | 0.034 | -0.074, 0.142 | -0.093 | -0.154, -0.033 | 0.11 |
| | | 16 or higher | 321 | 3.586 | 1.008 | -0.025 | -0.229, 0.179 | -0.011 | -0.140, 0.118 | 0.98 |
| | Model 2 | 11 or younger | 1,309 | 3.636 | 1.064 | 0.213 | 0.094, 0.331 | 0.053 | -0.014, 0.119 | 0.11 |
| | | 12-13 | 3,779 | 3.509 | 1.046 | Ref | | Ref | | NA |
| | | 14-15 | 1,854 | 3.496 | 1.031 | 0.025 | -0.079, 0.128 | -0.098 | -0.157, -0.038 | 0.12 |
| | | 16 or higher | 321 | 3.586 | 1.008 | -0.019 | -0.207, 0.168 | -0.035 | -0.161, 0.092 | 0.88 |
| | Model 3 | 11 or younger | 1,122 | 3.625 | 1.067 | 0.152 | 0.021, 0.284 | 0.067 | -0.003, 0.137 | 0.42 |
| | | 12-13 | 3,251 | 3.483 | 1.034 | Ref | | Ref | | NA |
| | | 14-15 | 1,580 | 3.462 | 1.021 | 0.00002 | -0.120, 0.120 | -0.083 | -0.145, -0.020 | 0.35 |
| | | 16 or higher | 272 | 3.547 | 1.002 | 0.062 | -0.280, 0.151 | -0.055 | -0.188, 0.077 | 0.96 |
| Glucose, (mmol/L)* | Model 1 | 11 or younger | 1,289 | 4.6 | 4.3, 4.9 | -0.721 | -2.422, 0.980 | 1.376 | 0.474, 2.277 | 0.11 |
| | | 12-13 | 3,741 | 4.5 | 4.3, 4.8 | Ref | | Ref | | NA |
| | | 14-15 | 1,832 | 4.6 | 4.3, 4.8 | 0.767 | -0.632, 2.165 | -0.429 | -1.237, 0.380 | 0.32 |
| | | 16 or higher | 322 | 4.6 | 4.3, 4.8 | -0.681 | -3.770, 2.407 | -0.840 | -2.550, 0.870 | 0.91 |
| | Model 2 | 11 or younger | 1,289 | 4.6 | 4.3, 4.9 | -0.291 | -1.936, 1.354 | 1.143 | 0.280, 2.006 | 0.25 |
| | | 12-13 | 3,741 | 4.5 | 4.3, 4.8 | Ref | | Ref | | NA |
| | | 14-15 | 1,832 | 4.6 | 4.3, 4.8 | 0.886 | -0.464, 2.235 | -0.317 | -1.090, 0.455 | 0.30 |
| | | 16 or higher | 322 | 4.6 | 4.3, 4.8 | -0.796 | -3.942, 2.349 | -0.917 | -2.553, 0.718 | 0.88 |
| | Model 3 | 11 or younger | 1,103 | 4.6 | 4.3, 4.9 | 0.008 | -1.986, 2.001 | 1.010 | 0.121, 1.890 | 0.44 |
| | | 12-13 | 3,217 | 4.5 | 4.3, 4.8 | Ref | | Ref | | NA |
| | | 14-15 | 1,563 | 4.6 | 4.3, 4.8 | 1.462 | -0.071, 2.994 | -0.288 | -1.083, 0.507 | 0.17 |
| | | 16 or higher | 272 | 4.6 | 4.3, 4.8 | -2.570 | -6.233, 1.093 | -0.752 | -2.434, 0.930 | 0.54 |
| Body-mass index (kg/m$^2$) | Model 1 | 11 or younger | 1,344 | 28.080 | 6.176 | 1.710 | 1.018, 2.401 | 1.848 | 1.481, 2.214 | 0.74 |
| | | 12-13 | 3,922 | 26.115 | 5.212 | Ref | | Ref | | NA |
| | | 14-15 | 1,929 | 25.061 | 4.767 | -0.456 | -0.961, 0.049 | -1.335 | -1.660, -1.009 | 0.02 |
| | | 16 or higher | 329 | 25.423 | 5.402 | -0.667 | -1.726, 0.392 | -1.057 | -1.752, -0.361 | 0.76 |

| | | | | | | | | | | |
|---|---|---|---|---|---|---|---|---|---|---|
| | Model 2 | 11 or younger | 1,344 | 28.080 | 6.176 | 1.653 | 0.970, 2.335 | 1.718 | 1.358, 2.078 | 0.84 |
| | | 12-13 | 3,922 | 26.115 | 5.212 | Ref | | Ref | | NA |
| | | 14-15 | 1,929 | 25.061 | 4.767 | -0.452 | -0.961, 0.057 | -1.330 | -1.650, -1.010 | 0.02 |
| | | 16 or higher | 329 | 25.423 | 5.402 | -0.672 | -1.724, 0.380 | -1.186 | -1.870, -0.503 | 0.65 |
| | Model 3 | 11 or younger | 1,122 | 3.625 | 1.067 | 1.736 | 0.985, 2.488 | 1.642 | 1.270, 2.014 | 0.93 |
| | | 12-13 | 3,251 | 3.483 | 1.035 | Ref | | Ref | | NA |
| | | 14-15 | 1,580 | 3.462 | 1.022 | -0.628 | -1.187, -0.069 | -1.237 | -1.569, -0.906 | 0.16 |
| | | 16 or higher | 272 | 3.547 | 1.002 | -1.429 | -2.545, -0.313 | -0.956 | -1.660, -0.251 | 0.55 |
| Waist circumference, (cm) | Model 1 | 11 or younger | 1,334 | 88.23 | 15.33 | 3.004 | 1.301, 4.707 | 3.513 | 2.559, 4.467 | 0.61 |
| | | 12-13 | 3,906 | 84.50 | 13.82 | Ref | | Ref | | NA |
| | | 14-15 | 1,920 | 82.54 | 12.69 | -0.767 | -2.171, 0.638 | -2.730 | -3.579, -1.881 | 0.06 |
| | | 16 or higher | 335 | 83.68 | 14.56 | -1.938 | -4.651, 0.776 | -1.621 | -3.427, 0.184 | 0.83 |
| | Model 2 | 11 or younger | 1,334 | 88.23 | 15.33 | 2.927 | 1.237, 4.616 | 3.155 | 2.217, 4.094 | 0.77 |
| | | 12-13 | 3,906 | 84.50 | 13.82 | Ref | | Ref | | NA |
| | | 14-15 | 1,920 | 82.54 | 12.69 | -0.775 | -2.181, 0.631 | -2.761 | -3.595, -1.926 | 0.06 |
| | | 16 or higher | 335 | 83.68 | 14.56 | -1.909 | -4.616, 0.797 | -2.018 | -3.794, -0.241 | 0.96 |
| | Model 3 | 11 or younger | 1,142 | 87.65 | 14.99 | 2.716 | 0.936, 4.498 | 3.056 | 2.085, 4.027 | 0.79 |
| | | 12-13 | 3,371 | 84.08 | 13.50 | Ref | | Ref | | NA |
| | | 14-15 | 1,634 | 81.95 | 12.15 | -1.266 | -2.745, 0.212 | -2.638 | -3.503, -1.773 | 0.22 |
| | | 16 or higher | 278 | 83.07 | 13.97 | -3.056 | -5.683, -0.429 | -1.656 | -3.495, 0.183 | 0.52 |

CI=confidence interval; HDL=high-density lipoprotein; SD=standard deviation.

Model 1 Adjusted for age
Model 2 Adjusted for age, qualifications, parental history of cardiovascular disease and parental history of diabetes. Blood pressure further adjusted for use of antihypertensive drugs, cholesterol levels adjusted for lipid lowering drugs and glucose adjusted for use of antidiabetic drugs.
Model 3 Adjusted for age, qualifications, parental history of cardiovascular disease, parental history of diabetes, smoking, alcohol intake and leisure time physical activity. Blood pressure further adjusted for use of antihypertensive drugs, cholesterol levels adjusted for lipid lowering drugs and glucose adjusted for use of antidiabetic drugs.

* Outcome log transformed and the coefficients reflect the percent change in the outcome.

**Table S12.** Association Between Age at Menarche and 10-Year Risk For Overall Cardiovascular Disease Excluding Individuals of Non-European ethnicity, the Scottish Family Health Study, 2006-2011.

| Risk score | Model | Age at menarche | N | Mean | SD | Within-sibships association | | Between-sibships association | | Bootstrap p-value for the difference in the within and between sibship association |
|---|---|---|---|---|---|---|---|---|---|---|
| | | | | | | β | 95% CI | β | 95% CI | |
| Framingham risk score * | Model 1 | 11 or younger | 1,051 | 26.07 | 0.90 | 0.10 | 0.02, 0.17 | 0.11 | 0.06, 0.15 | 0.80 |
| | | 12-13 | 2,956 | 25.94 | 0.90 | Ref | | Ref | | NA |
| | | 14-15 | 1,504 | 25.97 | 0.92 | 0.02 | -0.04, 0.08 | -0.03 | -0.07, 0.003 | 0.24 |
| | | 16 or higher | 267 | 26.07 | 0.86 | -0.03 | -0.15, 0.09 | 0.05 | -0.03, 0.13 | 0.48 |
| | Model 2 | 11 or younger | 1,051 | 26.07 | 0.90 | 0.09 | 0.01, 0.16 | 0.09 | 0.05, 0.13 | 0.88 |
| | | 12-13 | 2,956 | 25.94 | 0.90 | Ref | | Ref | | NA |
| | | 14-15 | 1,504 | 25.97 | 0.92 | 0.02 | -0.04, 0.08 | -0.04 | -0.07, -0.002 | 0.20 |
| | | 16 or higher | 267 | 26.07 | 0.86 | -0.03 | -0.15, 0.10 | 0.03 | -0.04, 0.10 | 0.63 |
| | Model 3 | 11 or younger | 902 | 26.04 | 0.90 | 0.08 | -0.01, 0.17 | 0.09 | 0.05, 0.13 | 0.88 |
| | | 12-13 | 2,538 | 25.92 | 0.89 | Ref | | Ref | | NA |
| | | 14-15 | 1,280 | 25.94 | 0.91 | 0.02 | -0.05, 0.09 | -0.02 | -0.06, 0.01 | 0.39 |
| | | 16 or higher | 227 | 26.02 | 0.83 | -0.04 | -0.18, 0.10 | 0.02 | -0.06, 0.09 | 0.66 |
| NHANES ECG risk equation score † | Model 1 | 11 or younger | 1,051 | 7.971 | 0.846 | 0.030 | -0.004, 0.064 | 0.018 | 0.001, 0.035 | 0.59 |
| | | 12-13 | 2,994 | 7.931 | 0.847 | Ref | | Ref | | NA |
| | | 14-15 | 1,544 | 7.976 | 0.882 | 0.005 | -0.023, 0.032 | -0.019 | -0.034, -0.004 | 0.24 |
| | | 16 or higher | 260 | 8.056 | 0.803 | 0.004 | -0.057, 0.065 | 0.024 | -0.008, 0.056 | 0.85 |
| | Model 2 | 11 or younger | 1,051 | 7.971 | 0.846 | 0.031 | -0.004, 0.065 | 0.016 | -0.002, 0.033 | 0.50 |
| | | 12-13 | 2,994 | 7.931 | 0.847 | Ref | | Ref | | NA |
| | | 14-15 | 1,544 | 7.976 | 0.882 | 0.006 | -0.022, 0.033 | -0.019 | -0.034, -0.004 | 0.22 |
| | | 16 or higher | 260 | 8.056 | 0.803 | 0.004 | -0.057, 0.066 | 0.022 | -0.010, 0.054 | 0.89 |
| | Model 3 | 11 or younger | 899 | 7.966 | 0.846 | 0.043 | 0.004, 0.082 | 0.023 | 0.006, 0.041 | 0.46 |
| | | 12-13 | 2,571 | 7.918 | 0.847 | Ref | | Ref | | NA |
| | | 14-15 | 1,306 | 7.935 | 0.866 | -0.016 | -0.048, 0.016 | -0.013 | -0.029, 0.003 | 1.00 |
| | | 16 or higher | 220 | 8.024 | 0.765 | -0.002 | -0.075, 0.071 | 0.024 | -0.008, 0.057 | 0.80 |

CI=confidence interval; ECG=electrocardiogram; SD=standard deviation.

* The variables included in the Framingham risk score is age, total cholesterol, HDL cholesterol, systolic blood pressure, smoking and diabetes.

† The information included in the NHANES ECG risk score included age, positive deflection of T axis, negative deflection of the T axis, heart rate and corrected QT interval.

Model 1 Adjusted for age

Model 2 Adjusted for age, qualifications, parental history of cardiovascular disease and parental history of diabetes.

Model 3 Adjusted for all of the covariates in Model 2 in addition to smoking (not adjusted for in the analysis of the Framingham risk score since part of the risk calculation), alcohol intake and leisure time physical activity.

**Table S13.** Association Between Age at Menarche and Cardiometabolic Health Outcomes Excluding Non-fasting Individuals, the Scottish Family Health Study, 2006-2011.

| Outcome | Model | Age at menarche | N | Mean/ Median | SD/Range | Within-sibships association | | Between-sibships association | | Bootstrap p-value for the difference in the within and between sibship association |
|---|---|---|---|---|---|---|---|---|---|---|
| | | | | | | β | 95% CI | β | 95% CI | |
| HDL cholesterol, (mmol/L) | Model 1 | 11 or younger | 1,139 | 1.543 | 0.411 | -0.075 | -0.127, -0.024 | -0.038 | -0.069, -0.008 | 0.37 |
| | | 12-13 | 3,275 | 1.591 | 0.414 | Ref | | Ref | | NA |
| | | 14-15 | 1,599 | 1.623 | 0.403 | -0.030 | -0.078, 0.018 | 0.039 | 0.011, 0.066 | 0.07 |
| | | 16 or higher | 274 | 1.631 | 0.429 | -0.027 | -0.117, 0.062 | 0.048 | -0.009, 0.105 | 0.30 |
| | Model 2 | 11 or younger | 1,139 | 1.543 | 0.411 | -0.077 | -0.128, -0.025 | -0.029 | -0.059, 0.001 | 0.24 |
| | | 12-13 | 3,275 | 1.591 | 0.414 | Ref | | Ref | | NA |
| | | 14-15 | 1,599 | 1.623 | 0.403 | -0.031 | -0.079, 0.017 | 0.039 | 0.012, 0.066 | 0.06 |
| | | 16 or higher | 274 | 1.631 | 0.429 | -0.028 | -0.118, 0.062 | 0.057 | 0.001, 0.114 | 0.25 |
| | Model 3 | 11 or younger | 971 | 1.556 | 0.411 | -0.065 | -0.125, -0.006 | -0.024 | -0.055, 0.007 | 0.39 |
| | | 12-13 | 2,809 | 1.602 | 0.411 | Ref | | Ref | | NA |
| | | 14-15 | 1,367 | 1.635 | 0.408 | -0.040 | -0.095, 0.015 | 0.042 | 0.014, 0.069 | 0.05 |
| | | 16 or higher | 272 | 1.652 | 0.431 | -0.045 | -0.143, 0.054 | 0.054 | -0.004, 0.112 | 0.21 |
| Non-HDL cholesterol (mmol/L) | Model 1 | 11 or younger | 1,139 | 3.630 | 1.058 | 0.202 | 0.064, 0.341 | 0.047 | -0.025, 0.119 | 0.18 |
| | | 12-13 | 3,275 | 3.530 | 1.050 | Ref | | Ref | | NA |
| | | 14-15 | 1,599 | 3.511 | 1.021 | 0.059 | -0.066, 0.184 | -0.099 | -0.163, -0.034 | 0.08 |
| | | 16 or higher | 274 | 3.589 | 1.006 | -0.057 | -0.302, 0.188 | -0.028 | -0.163, 0.107 | 0.92 |
| | Model 2 | 11 or younger | 1,139 | 3.630 | 1.058 | 0.204 | 0.072, 0.337 | 0.033 | -0.038, 0.103 | 0.11 |
| | | 12-13 | 3,275 | 3.530 | 1.050 | Ref | | Ref | | NA |
| | | 14-15 | 1,599 | 3.511 | 1.021 | 0.046 | -0.075, 0.168 | -0.101 | -0.164, -0.039 | 0.10 |
| | | 16 or higher | 274 | 3.589 | 1.006 | -0.043 | -0.267, 0.182 | -0.044 | -0.176, 0.088 | 0.98 |
| | Model 3 | 11 or younger | 971 | 3.619 | 1.062 | 0.155 | 0.003, 0.306 | 0.042 | -0.032, 0.116 | 0.34 |
| | | 12-13 | 2,809 | 3.510 | 1.042 | Ref | | Ref | | NA |
| | | 14-15 | 1,367 | 3.472 | 1.014 | 0.055 | -0.083, 0.193 | -0.104 | -0.170, -0.038 | 0.11 |
| | | 16 or higher | 234 | 3.560 | 1.009 | -0.075 | -0.328, 0.178 | -0.046 | -0.185, 0.093 | 0.96 |
| Glucose, (mmol/L)* | Model 1 | 11 or younger | 1,125 | 4.6 | 4.3, 4.9 | -1.351 | -2.916, 0.213 | 1.229 | 0.398, 2.059 | 0.04 |
| | | 12-13 | 3,248 | 4.5 | 4.3, 4.8 | Ref | | Ref | | NA |
| | | 14-15 | 1,583 | 4.6 | 4.3, 4.8 | -0.095 | -1.577, 1.387 | -0.504 | -1.246, 0.238 | 0.80 |
| | | 16 or higher | 275 | 4.6 | 4.3, 4.8 | -0.560 | -4.375, 3.256 | -0.589 | -2.153, 0.975 | 0.87 |

| | | | | | | | | | | |
|---|---|---|---|---|---|---|---|---|---|---|
| | Model 2 | 11 or younger | 1,125 | 4.6 | 4.3, 4.9 | -0.865 | -2.382, 0.653 | 1.209 | 0.398, 2.020 | 0.10 |
| | | 12-13 | 3,248 | 4.5 | 4.3, 4.8 | Ref | | Ref | | NA |
| | | 14-15 | 1,583 | 4.6 | 4.3, 4.8 | 0.006 | -1.385, 1.398 | -0.294 | -1.019, 0.430 | 0.81 |
| | | 16 or higher | 275 | 4.6 | 4.3, 4.8 | -0.682 | -4.505, 3.141 | -0.437 | -1.964, 1.090 | 0.80 |
| | Model 3 | 11 or younger | 958 | 4.6 | 4.3, 4.9 | -0.263 | -1.966, 1.440 | 1.087 | 0.244, 1.930 | 0.29 |
| | | 12-13 | 2,785 | 4.5 | 4.3, 4.8 | Ref | | Ref | | NA |
| | | 14-15 | 1,355 | 4.6 | 4.3, 4.8 | 0.479 | -1.024, 1.983 | -0.332 | -1.084, 0.420 | 0.54 |
| | | 16 or higher | 234 | 4.6 | 4.3, 4.8 | -3.115 | -7.624, 1.394 | -0.155 | -1.740, 1.430 | 0.41 |

CI=confidence interval; HDL=high-density lipoprotein; SD=standard deviation.

Model 1 Adjusted for age
Model 2 Adjusted for age, ethnicity, qualifications, parental history of cardiovascular disease and parental history of diabetes. Blood pressure further adjusted for use of antihypertensive drugs, cholesterol levels adjusted for lipid lowering drugs and glucose adjusted for use of antidiabetic drugs.
Model 3 Adjusted for age, ethnicity qualifications, parental history of cardiovascular disease, parental history of diabetes, smoking, alcohol intake and leisure time physical activity. Blood pressure further adjusted for use of antihypertensive drugs, cholesterol levels adjusted for lipid lowering drugs and glucose adjusted for use of antidiabetic drugs.

[*] Outcome log transformed and the coefficients reflect the percent change in the outcome.
